# Lysophosphatidic Acid Receptor 6 (LPAR6) Is a Potential Biomarker Associated with Lung Adenocarcinoma

**DOI:** 10.3390/ijerph182111038

**Published:** 2021-10-20

**Authors:** Jian He, Rui Gao, Mei Meng, Miao Yu, Chengrong Liu, Jingquan Li, Yizhi Song, Hui Wang

**Affiliations:** 1 State Key Laboratory of Oncogenes and Related Genes, Center for Single-Cell Omics, School of Public Health, Shanghai Jiao Tong University School of Medicine, Shanghai 200025, China; 184163@shsmu.edu.cn (R.G.); mm2020@sjtu.edu.cn (M.M.); jqli@shsmu.edu.cn (J.L.); 2 CAS Key Laboratory of Bio-Medical Diagnostics, Suzhou Institute of Biomedical Engineering and Technology, Chinese Academy of Sciences, Suzhou 215163, China; ym0920@mail.ustc.edu.cn; 3 Shanghai Institute of Immunology, Department of Immunology and Microbiology, Shanghai Jiao Tong University School of Medicine, Shanghai 200025, China; chengrong@sjtu.org

**Keywords:** LPAR6, lung adenocarcinoma, biomarker, tumor infiltration lymphocytes, prognosis

## Abstract

LPAR6 is the most recently determined G-protein-coupled receptor of the lysophosphatidic acid receptor, and very few of studies have demonstrated the performance of LPAR6 in cancers. Moreover, the relationship of LPAR6 to the potential of prognosis and tumor infiltration immune cells in different types of cancer are still unclarified. In this study, the mRNA expression of LPAR6 and its clinical characteristics were evaluated on various databases. The association between LPAR6 and immune infiltrates of various types of cancer were investigated via TIMER. Immunohistochemistry (IHC) for LPAR6 in lung adenocarcinoma (LUAD) and lung squamous cell carcinoma (LUSC) tissue microarray with patients’ information was detected. We constructed a systematic prognostic landscape in a variety of types of cancer base on the expression level of mRNA. We enclosed that higher LPAR6 mRNA expression level was associated with better overall survival in some types of malignancy. Moreover, LPAR6 significantly affects the prognostic potential of various cancers in The Cancer Genome Atlas Program (TCGA), especially in lung cancer. Tissue microarrays of lung cancer patient cohorts demonstrated that a higher protein level of LPAR6 was correlated to better overall survival of LUAD rather than LUSC cohorts. Further research indicated that the underlying mechanism of this phenome might be the mRNA expression level of LPAR6 was positively associated to infiltrating statuses of devious immunocytes in LUAD rather than in LUSC, that is, LPAR6 expression potentially contributes to the activation and recruiting of T cells (CD8+ T, naive T, effector T cell) and NK cells and inactivates Tregs, decreases T cell exhaustion and regulates T-helper (Th) cells in LUAD. Our discovery implies that LPAR6 is associated with prognostic potential and immune-infiltrating levels in LUAD. These discoveries imply that LPAR6 could be a promising novel biomarker for indicating the prognosis potential of LUAD patients.

## 1. Introduction

Lung cancer is one of the most common malignancies of all incident cases in both men and women around the world, and metastasis is the crucial biological procedure leading to a poor prognosis [1]. It is the top diagnosed malignancy in China and the second most common malignancy in the U.S., and is also the main reason of cancer-related deaths both in China and the U.S. [2]. Scientists have made great efforts to treat various types of lung cancer, but there is still a large amount of time and effort left to do. According to histopathological classification, lung cancer could be classified as two broad subtypes, small cell lung cancer (SCLC) and non-small-cell lung cancer (NSCLC), and the NSCLC is more prevalent [3]. Lung squamous cell carcinoma (LUSC) and lung adenocarcinoma (LUAD) are the top two most common subtype NSCLC [4]. Surgery is the primary treatment option during the early stages of the NSCLC, while in late stages, surgery is combined with chemotherapies, and/or radiotherapy [4]. However, though these treatment procedures were applied, the prognosis of the patients remains poor, and also the post-treatment recurrence is the main cause of the disease, where the total 5-year survival rate for all stages is only 16.6% [4].

NSCLC has been regarded as a kind of non-immunogenic disease in the past 20 years. However, more and more knowledge of tumor immune interactivities has opposed this model in lung cancer and some other types of malignancy. Immune-related interaction mechanisms act as a crucial role in oncogenesis and development, and immune therapy is considered a promising approach for cancer treatment [4,5], based on this, scientists are trying to employ the body’s own immune system to resist and defeat malignancies [6]. Recently, immunotherapies, including adoptive cell transfers, monoclonal antibodies and vaccines, have become more and more applied to the clinic therapeutics of many types of cancer, such as melanoma, and most recently for lung cancer [7]. In recent years, the finding of antibodies that target the immune checkpoints has revolutionized the treatment of NSCLC, including programmed cell death protein-1 (PD-1) and programmed cell death protein ligand-1 (PD-L1) [8], and these two therapeutic approaches above have showed promising anti-tumor performance in melanoma and NSCLC [9,10,11]. In addition, more and more research has demonstrated that tumor-infiltrating lymphocytes (TILs) play a crucial role in modulating the response to chemotherapy and heighten the clinical prognosis potential of various types of cancer [12,13], for example, tumor-infiltrating neutrophils (TINs) [14,15,16] and tumor-associated macrophages (TAMs), they also associate with the prognosis [17,18,19,20]. So, it is an essential and urgent requirement to elucidate the immunophenotypes of tumor immune interactivities and the identification of new immune therapy targets for lung cancer patients.

LPA is a kind of lipid that is involved in the proliferation of tumor cells via its G-protein coupled (GPC) receptors [21,22] and one of their receptors—LPAR6—is the latest identified receptor of LPA [23,24]. Moreover, it has been demonstrated to be related to many types of tumors, including prostate [25], liver [26,27], colorectal [28,29] and pancreatic cancer [30]. However, the function of LPAR6 remains highly controversial since in colorectal cancer, scientists found that LPAR6 acts as a tumor suppressor, whereas it acts as a facilitator in the other types of tumors [25,26,27,30,31]. All these indicate that LPAR6 plays an important role in cancer, whereas the relationship between LPAR6 and tumor biology and the underlying mechanism involved is still unclear.

Here, we investigated the mRNA expression level of LPAR6 and the correlation with prognosis patterns of cancer patients in databases using bioinformatics tools. In addition, we analyzed the correlation of LPAR6 with tumor-infiltrating immune cells (TIICs) in various tumor microenvironments via TIMER. Moreover, IHC staining for LPAR6 in two separate lung cancer cohorts with patients’ information was detected to analyze the correlation of the expression of LPAR6 and the clinicopathological parameters of lung cancer.

All these discoveries indicated the crucial role of LPAR6 in lung cancer and also provided a potential correlation and identified the mechanism involved between LPAR6 and tumor–immune interactions.

## 2. Materials and Methods

### 2.1. Ethics Approval

This project was permitted and under the supervision of Ethics Committee of Shanghai Jiao Tong University School of Medicine.

### 2.2. LPAR6 Gene Expression Level Analysis

The mRNA expression level of the LPAR6 in various types of cancer was investigated via different database, including Oncomine, TIMER and GEPIA2 [32]. The threshold in Oncomine database was as follows: *p*-value of 10^−4^, fold change of 1.5, and gene ranking top 5%.

### 2.3. Prognosis Analysis

The association between LPAR6 mRNA expression level and survival rate in different types of cancer was investigated using the PrognoScan and GEPIA2 database, which searching for relationships between gene expression and the prognoses of patients, such as overall survival (OS) and disease-free survival (DFS), across a large collection of microarray datasets [33,34]. The threshold was adjusted to a Cox *p*-value < 0.05.

### 2.4. Correlation Analysis of LPAR6 and Survival Rate

The correlation between the mRNA expression level of LPAR6 and survival rate as well as different cancer staging in various cancers was determined by Kaplan-Meier plotter database [35]. The HRs with 95% confidence intervals (Cls) and log-rank *p*-value were analyzed.

### 2.5. Methylation Analysis

UALCAN [36] could be employed to investigate methylation and mRNA expression levels, also the survival of a specific target gene across several clinicopathological features, such as stages and age. The *t*-test was performed to compare the statistical significance.

### 2.6. Biological Network Analysis

GeneMANIA is identified single genes related to a set of input genes [37] to construct the LPAR6 biological network based on a set of function-association data, including co-expression, genetic and protein interaction pathways, colocalization and protein domain homology.

### 2.7. LinkedOmics Analysis

Thirty-two types of cancer and over 10,000 patients from TCGA were included in the LinkedOmics database [38]. LinkFinder was employed to determine the differentially expressed genes (DEGs) in TCGA. LUAD and LUSC cohorts whose expression levels correlated with those of LPAR6. These results were investigated by using Pearson’s correlation coefficient. LinkInterpreter was employed to identify the pathways and networks [39].

### 2.8. Immune Infiltrates Level and Gene Correlation Analysis

We investigated the mRNA expression level of LPAR6 in various types of malignancy and the association of LPAR6 level the abundance of immune-infiltrating, including various types of T cells (CD4+ T cells, CD8+ T cells), B cells, macrophages, neutrophils, and DCs, via gene modules in TIMER database [40,41,42,43]. In addition, associations between LPAR6 expression level and marker genes of TIICs were explored via correlation modules. The marker genes of TIICs included markers of T cells (CD8+ T cells, general T cells), B cells, TAMs, monocytes, macrophages (type 1 macrophages, M1 and Type 2 macrophages, M2), neutrophils, natural killer (NK) cells, dendritic cells (DCs), T-helper cells (Th1, Th2 and Th17), follicular helper T cells (Tfh), T regulatory cells (Tregs) and exhausted T cells. The gene marker sets are referenced in our previous publication [44,45]. The expression level of the genes was demonstrated by using log2 RSEM.

### 2.9. Immunohistochemical Staining for LPAR6 in Lung Cancer Patient Cohort Tissue Microarrays

Here, two tissue microarrays were constructed using formalin-fixed paraffin-embedded (FFPE) tissue samples from LUAD (LUC1601) and LUSC (LUC1602) patients, each tissue microarrays (TMA) chip containing 74 and 78 paired tumor and adjacent normal tissues were purchased from the Superbiotek Co., Ltd., (Shanghai, China), respectively. Clinicopathological data including subtype, histological grading, and tumor/nodal stage and information about patient follow-up could be retrieved from the database of the Shanghai Jiao Tong University School of Medicine.

The tissue sections underwent immunohistochemical staining using a primary antibody to LPAR6 (Thermo Fisher/Invitrogen, Waltham, MA, USA) (Cat No. PA5-33901) at a dilution of 1: 100. Sections of the TMAs were used for investigating the protein level of LPAR6 following the general standard IHC staining protocols.

### 2.10. Statistical Analysis

The results produced via Oncomine as mentioned in Section 2.1. The consequence of Kaplan–Meier plots, GEPIA, and PrognoScan are exhibited with HR and *p* or Cox *p*-values from a log-rank test. Further, the correlation coefficient of gene expression was evaluated by Spearman’s correlation and *p*-values < 0.05 were considered statistically significant. Protein level was determined by the staining intensity and the distribution of the positive cells, which were performed by two independent pathologists blinded to the clinical information of the patients as described [45].

## 3. Results

### 3.1. The Expression Levels of LPAR6 in Different Human Cancers

**Figure 1:**
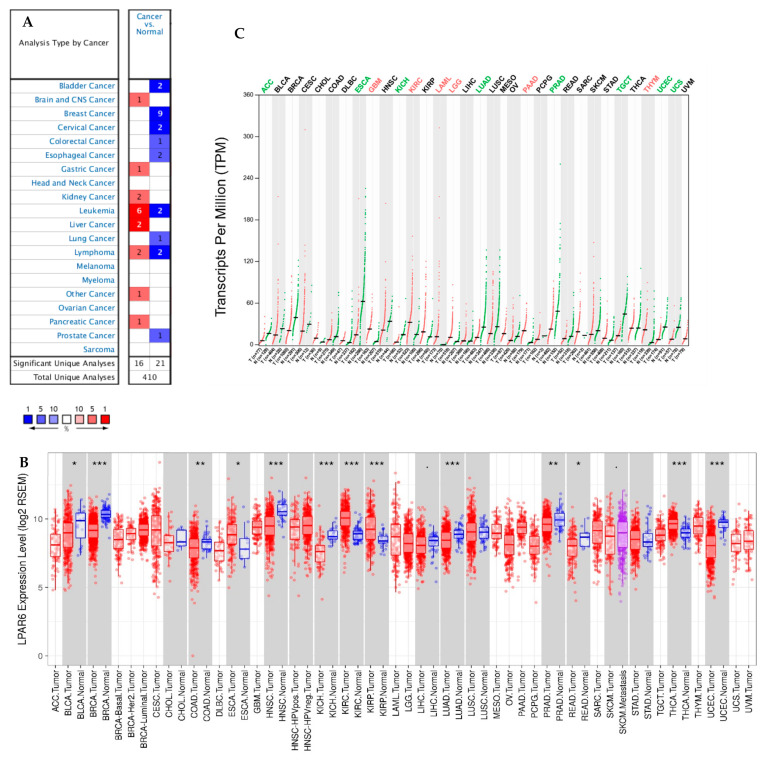
LPAR6 mRNA expression levels in different types of human cancers in different databases. (**A**) Increased or decreased LPAR6 in data sets of different cancers compared with normal tissues. Cell color is determined by the best gene rank percentile for the analyses within the cell. (**B**) Human *LPAR6* expression levels in different tumor types from TCGA database. One category of cancer is in one box, and paired tissue (tumor and adjacent) are in grey boxes. *p* < 0.1, * *p* < 0.05, ** *p* < 0.01, *** *p* < 0.001. (**C**) *LPAR6* expression profile across all tumor samples and paired normal tissues (Dot plot) via GEPIA. Each dots represent the expression of samples.

To investigate the varied mRNA expression level of LPAR6 in tumor and normal tissues, the LPAR6 expression levels were determined using the dominant online database, Oncomine and GEPIA2. Here, we enclosed the LPAR6 expression was higher in brain and central nervous system (CNS) cancer, gastric, liver cancer, kidney, lymphoma and pancreatic cancer compared to the normal tissues and lower mRNA expression level of LPAR6 was observed in breast, bladder, colorectal, cervical, lung, esophageal, prostate cancer and some other types of cancer compared to the adjacent normal tissues (cancer vs. normal) (Figure 1A). This detail of the expression level of LPAR6 in various types of cancer is summarized in Supplementary Table S1. To evaluate the mRNA expression level of LPAR6 in cancers, we studied the levels of LPAR6 expression employing the RNA-Seq datasets in the TCGA. The varied expression levels between tumor and adjacent normal tissues for LPAR6 across each type of TCGA tumors is demonstrated in Figure 1B. The expression level of LPAR6 was significantly lower in the tumor tissue of bladder urothelial carcinoma (BLCA), breast invasive carcinoma (BRCA), colon adenocarcinoma (COAD), head and neck cancer (HNSC), kidney chromophobe (KICH), LUAD, prostate adenocarcinoma (PRAD), rectum adenocarcinoma (READ) and uterine corpus endometrial carcinoma (UCEC) compared with adjacent normal tissues and was significantly higher in esophageal carcinoma (ESCA), kidney renal clear cell carcinoma (KIRC), kidney renal papillary cell carcinoma (KIRP), thyroid carcinoma (THCA) compared with the normal tissues (Figure 1B). GEPIA2 generates dot plots to profile gene/isoform expression across various types of cancer and paired normal tissue samples, and each dot representing a distinct sample. The differential mRNA expression level of LPAR6 between tumor and matched normal and GTEx data across all TCGA tumors by GEPIA2 are demonstrated in Figure 1C. LPAR6 expression was significantly higher in glioblastoma multiforme (GBM), KIRC, LAML, lower grade glioma (LGG), PAAD, thymoma (THYM) and lower in adrenocortical carcinoma (ACC), ESCA, KICH, LUAD, PRAD, testicular germ cell tumors (TGCT), UCEC and UCS compared with normal GTEx tissues. From these above, we found that the expression patterns are different in two types of lung cancers, LUAD and LUSC.

### 3.2. Prognostic Potential of LPAR6 in Various Types of Cancer

**Figure 2:**
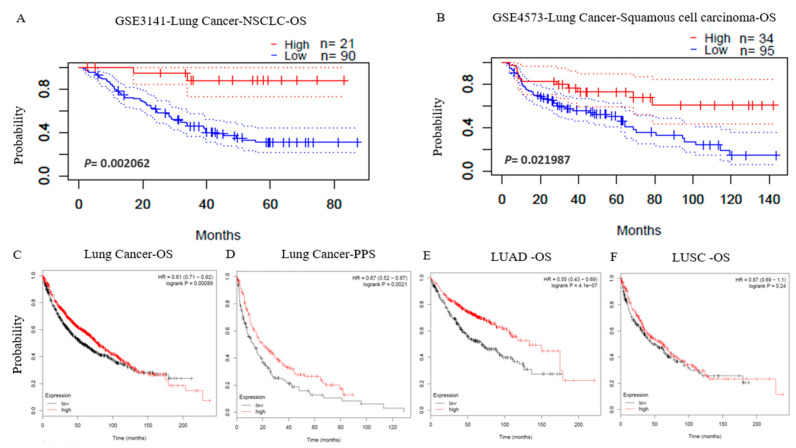
Kaplan–Meier survival curves comparing the high and low expression of LPAR6 in different types of cancer in the PrognoScan and Kaplan–Meier plotter databases. (**A**,**B**) Survival curves of OS in two lung cancer cohorts [GSE3141 (*n* = 111, *p* = 0.00206181) and GSE4573 (*n* = 129, *p* = 0.0219869)] and DSS in bladder cancer cohort [GSE13507 (*n* = 165, *p* = 0.0067285)]. (**C**,**D**) Survival curves of OS (*n* = 1926) and PPS (*n* = 344) of the lung cancer. (**E**,**F**) Survival curves of OS of the lung adenocarcinoma (*n* = 720) and squamous cell carcinoma (*n* = 524).

We analyzed whether the mRNA expression level of LPAR6 was associated with the prognosis specific across cancer patient cohorts. The effects of LPAR6 mRNA expression on the various survival rates were assessed using PrognoScan. The detailed relationship between the mRNA expression level of LPAR6 and Prognostic potential of various cancers are listed in Supplementary Table S2. Notably, the expression level of LPAR6 impacts OS in lung cancer significantly (Figure 2A,C). Two cohorts (GSE3141 and GSE4573) of lung cancer demonstrated that high expression level of LPAR6 was associated with better prognosis (OS HR = 0.53, 95% CI = 0.36 to 0.80, Cox *p* = 0.00206181; OS HR = 0.53, 95% CI = 0.31 to 0.91, Cox *p* = 0.0219869). (Figure 2A,B), and better post-progression survival (PPS) (Figure 2D). So, it is conceivable that high LPAR6 expression is an independent risk factor and leads to a better prognosis in lung cancer patients, and a hazard ratio (HR) below 0 indicates LPAR6 expression is a protective factor. Additionally, high LPAR6 expression significantly impacts disease-specific survival (DSS) in bladder cancer (Supplementary Figure S1A) and OS (Supplementary Figure S1B,M), relapse-free survival (RFS) (Supplementary Figure S1C), DSS (Supplementary Figure S1D,H), distant metastasis-free survival (DMFS) (Supplementary Figure S1N) in breast cancer. Moreover, three cohorts (GSE19615, GSE9195 and GSE11121) of breast cancer demonstrated that higher expression level of LPAR6 was correlated with a better prognostic potential of DMFS (Supplementary Figure S1E–G). Further, a higher LPAR6 expression level was associated with better prognosis potential in some other types of cancer (Supplementary Figure S1I–K).

In addition to microarray analysis data of LPAR6, the RNA-Seq was also used to analyze the prognosis of LPAR6 in various types of cancers via the same database. A better prognosis in liver cancer is shown to be associated with a higher LPAR6 expression level (Supplementary Figure S1O–Q). The different correlation patterns between adenocarcinoma and squamous cell carcinoma of lung cancer attracted our attention (Figure 2E,F). These data confirmed the prognostic value of LPAR6 in some specific types of cancers, that is, the increased or decreased LPAR6 expression has different prognostic values depending on the type of cancers.

### 3.3. The mRNA Expression Level of LPAR6 Impacts the Lung Cancer Prognosis in Different Clinical Characteristics

Here we studied the association with the expression level of LPAR6 and different clinical characteristics (stages and treatments) in order to better disclosure the mechanisms and relevance of the mRNA expression level of LPAR6 in different types of cancer, especially in different clinical stages of lung cancer patients.

We found that high mRNA expression level of LPAR6 was associated with better OS only in Stage 1 and 2 of LUAD (OS HR = 0.27, *p =* 4.6 × 10^−10^; OS HR = 0.51, *p =* 0.0073) not in LUSC (Table 1). This arrestive phenomenon combines with the different survival patterns of LUAD and LUSC in Figure 2E,F may indicate the correlation of LPAR6 expression and the prognosis of different types of cancer depends on the different mechanisms in the carcinogenesis and development.

### 3.4. Low Promoter Methylation Levels of LPAR6 Impacts the Clinicopathological Parameters of Lung Cancer Patients

**Figure 3:**
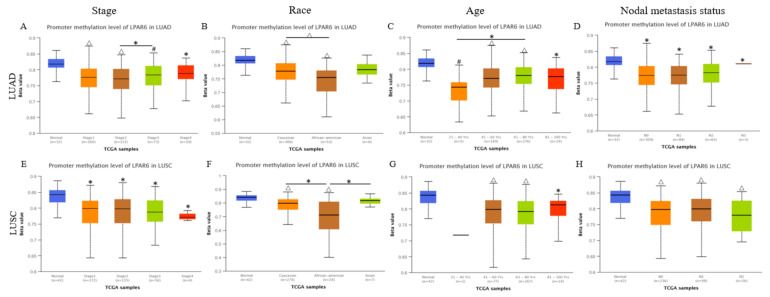
Promoter methylation levels of LPAR6 impacts the clinicopathological parameters. (**A**,**E**) stage, (**B**,**F**) race, (**C**,**G**) age, (**D**,**H**) nodal metastasis status in LUAD and LUSC cohorts respectively. * *p* < 0.05 compare with the normal tissue, △ *p* < 0.05 compare within different group in the tumor tissue.

The lower promoter methylation levels of LPAR6 were detected in the earlier stage, implying that lower promoter methylation levels of LPAR6 were correlated with the earlier stages of the progress of lung cancer (Figure 3). We also found that late-stage (stage 4) with the lowest promoter methylation showed levels of LPAR6 in LUSC whereas it is the highest methylation level in entire cancer progress in LUAD cohorts (stage 1–4) (Figure 3A,E), and the lowest promoter methylation levels of LPAR6 appears in an earlier stage (stage 2) of LUAD while in the late stage of LUSC. In addition, the results of promoter methylation here could indicate how LPAR6 expression levels fluctuate in various stages of lung cancer. What interested us is that the same pattern was detected in nodal metastasis analysis, which implies that in the later stage, the promoter methylation levels of LPAR6 are correlated with nodal metastasis in some way (Figure 3D,H). The promoter methylation levels of LPAR6 share a similar pattern in LUAD and LUSC among different races and different ages, respectively; that is, both in the African-American group and younger group of these two cohorts had the lowest promoter methylation levels of LPAR6 (Figure 3B,F).

### 3.5. Interaction Network of LPAR6

**Figure 4:**
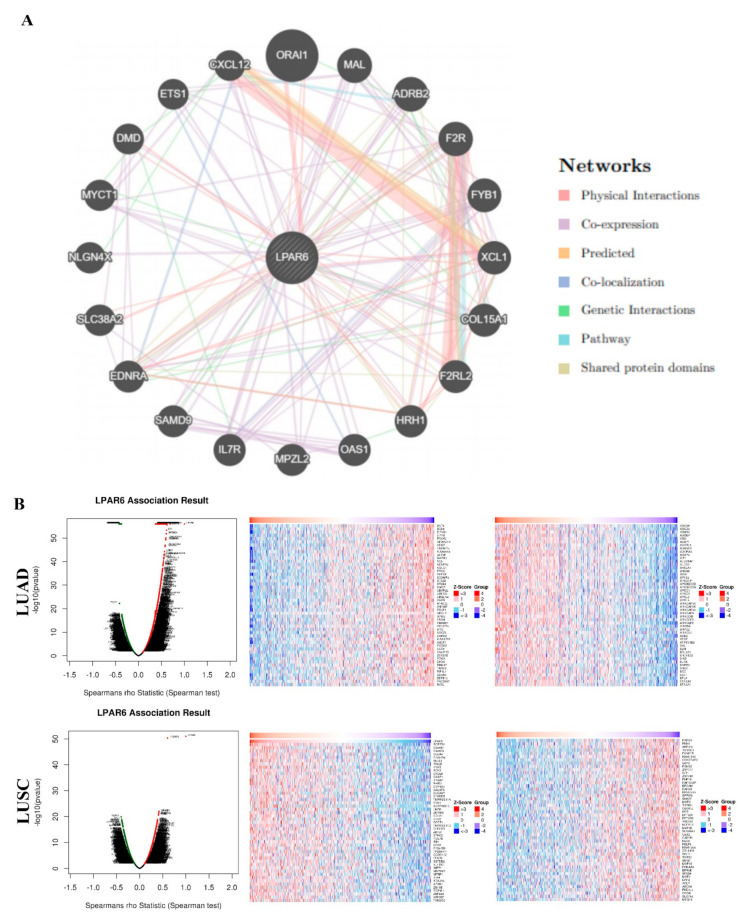

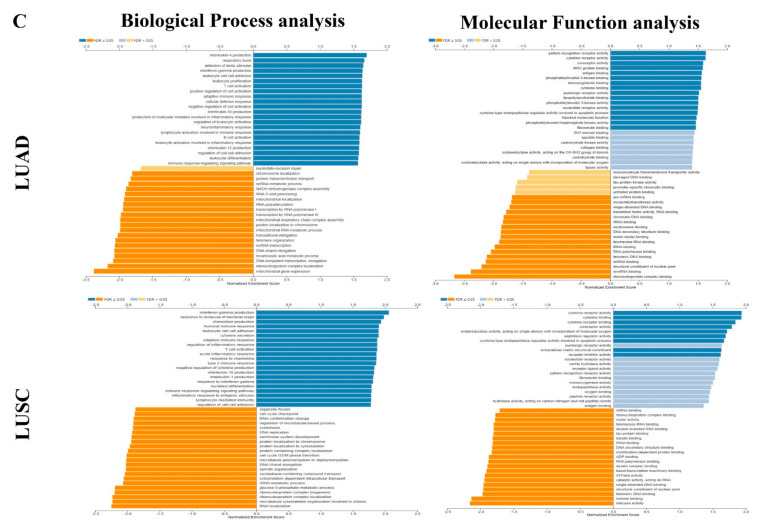
Biological interaction network of LPAR6. LPAR6 interaction network in TCGA, different colors represent diverse bioinformatics methods (**A**) and differentially expressed genes in correlation with LPAR6 and heat maps of positively and negatively correlated genes with LPAR6 in LUAD and LUSC were analyzed by Pearson test (**B**). Enriched gene ontology annotations of biological process and molecular function analysis of LPAR6 correlated genes in LUAD and LUSC (**C**). Red indicates positive and blue indicates negative. Dark blue and orange indicate FDR ≤ 0.05, light blue and orange indicate FDR > 0.05. FDR, false discovery rate.

An interaction network of LPAR6 was constructed to determine potential interactions between LPAR6 and other cancer-associated proteins. These data demonstrated that LPAR6 has co-expression with 19 proteins, shared protein domains with ADRB2 and physical interactions with DMD (dystrophin) (Figure 4A). Moreover, the analysis of interactions network of LRP6 with the 19 protein and its impact on lung cancer progression in Human Protein Atlas demonstrated that the “19 protein set” associate to a better prognosis in LUAD rather than that of LUSC (Supplementary Figure S2). Firstly, we found some proteins in the “19 protein set” are significantly positively correlated with the prognosis in LUAD patients, while these genes are significantly negatively correlated with the prognosis in LUSC patients, including MAL (T cell differentiation protein), CXCL12 (C-X-C motif chemokine ligand 12), ADRB2 (Adrenoceptor beta 2). Secondly, the protein level of DMD (Dystrophin) and IL7R (Interleukin 7 receptor) showed a significantly positive pattern in LUAD cohorts rather than that of LUSC cohorts. Thirdly, the protein level of MYCT1 (MYC target 1), EDNRA (Endothelin receptor type A) and F2R (Coagulation factor II thrombin receptor) demonstrated a significantly negative pattern in LUSC patients rather than that of LUAD patients (SI-Figure S2). All these above indicate that the protein level of LPAR6 and the “19 protein set” associate to a better prognosis in LUAD rather than that of LUSC (Supplementary Figure S2). LinkedOmics were then used to analyze the genes that co-expressed with LPAR6 in lung cancers. The volcano plot elucidated that the expression of genes was negatively correlated with that of LPAR6 (green spot; false discovery rate (FDR) < 0.05), while genes expression is positively correlated with LPAR6 (red spot; FDR < 0.05; Figure 4B). The top 50 positively and negatively correlated genes are showed in Figure 4B. These results imply that LPAR6 serves an important role in cancer development. Biological process and molecular function studies were conducted using gene set enrichment analysis, which showed that LPAR6-associated DEGs were involved in several kinds of immune biology process such as ‘interleukin production’, ‘respiratory burst’, ‘leukocyte proliferation’, ‘T cell activation’, ‘adaptive immune response’ were involved in LUAD and LUSC, respectively (Figure 4C). All these data imply that LPAR6 may serves a key role in immune system activation, metabolism, cellular responses to stimulation and many other processes.

### 3.6. The Expression Level of LPAR6 Is Correlated with Immune Infiltration Level in Lung Cancers

TILs have been proved as an independent predictor of survival in different types of cancer [46,47]. So, in this study, we determined whether the expression level of LPAR6 correlates with the immune-infiltration levels in various types of cancer. We analyzed the correlations of LPAR6 mRNA expression with the immune-infiltration levels in nearly 40 types of cancer. The results show that the mRNA expression level of LPAR6 has significant negative correlations with tumor purity in 26 types of cancer which indicating LPAR6 somehow related to recruiting lymphocytes to tumor and significant correlated with B cell infiltration levels in 13 types of cancers. In addition, the expression level of LPAR6 has significant correlations with infiltrating levels of CD8+ T cells in 24 types of cancer, CD4+ T cells in 26 types of cancer, neutrophils in 33 types of cancer, macrophages in 20 types of cancer and dendritic cells in 21 types of cancer (Supplementary Table S3 and Figure S3).

Given the correlation of the mRNA expression level of LPAR6 with immune-infiltration level in diverse types of cancer, we investigated the distinct types of cancer in which LPAR6 was correlated with prognosis and immune-infiltration. Tumor purity is a crucial factor that influences the analysis of immune-infiltration in clinical tumor samples by genomic approaches [34,41]. So here we selected the cancer types in which LPAR6 expression levels have a significant negative correlation with tumor purity in TIMER and a significant correlation with prognosis. Interestingly, we found that the expression level of LPAR6 expression correlates with better OS and high immune-infiltration levels in breast cancer, liver cancer and LUAD rather than in LUSC.

**Figure 5:**
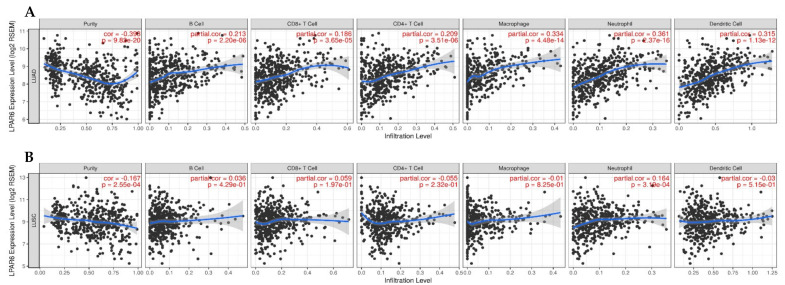
Correlation of LPAR6 expression with immune infiltration level in (**A**) LUAD, (**B**) LUSC. (**A**) LPAR6 expression is significantly negatively related to tumor purity and has significant strong positive correlations with the level of B cells, CD8+ T cells, macrophages, neutrophils, and DCs in LUAD (*n* = 515). (**B**) LPAR6 expression is significantly negatively related to tumor purity and has weak positive correlations with infiltrating levels of neutrophils in LUSC but no significant correlation with infiltrating levels of B cells, CD8+ T cells, macrophages and DCs (*n* = 501).

The LPAR6 expression level of LUAD and LUSC are all significantly negatively related to tumor purity (Figure 5). LPAR6 mRNA expression level has significant positive correlations with the infiltrating levels of B cell, CD4+ T cells, CD8+ T cell, macrophages, neutrophils and DCs in LUAD (Figure 5). What interested us is that the correlation with immune cells demonstrated a different pattern in LUAD and LUSC of lung cancers. These findings strongly suggest that LPAR6 plays a specific role in immune-infiltration in different types of lung cancer, and leads to a better prognosis in LUAD instead of in LUSC.

### 3.7. Correlation Analysis between LPAR6 Expression Level and the Immune Marker Sets

**Figure 6:**
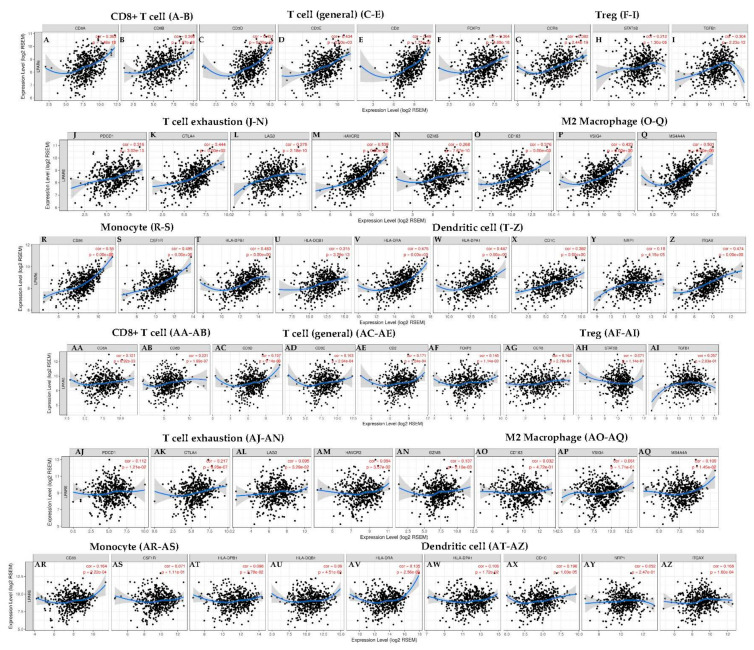
Correlation analysis between LPAR6 expression and immune marker sets in LUAD and LUSC. Markers include CD8A and CD8B of CD8+ T cell; CD3D, CD3E and CD2 of general T cell; FOXP3, CCR8, STAT5B and TGFB1 of Treg; PDCD1, CTLA4, LAG3, HAVCR2 and GZMB of exhausted T cells; CD163, VSIG4 and MS4A4A of M2 macrophages; CD86 and CSF1R of monocytes; HLA-DPB1, HLA-DQB1, HLA-DRA, HLA-DPA1, CD1C, NRP1 and ITGAX of Dendritic cell. (**A**–**Z**) Scatterplots of correlations between LPAR6 expression and gene markers of CD8+ T cell (**A**,**B**), general T cell (**C**–**E**), Treg (**F**–**I**), T cell exhaustion (**J**–**N**), M2 macrophage (**O**–**Q**), monocyte (**R**–**S**) and dendritic cell (**T**–**Z**) in LUAD. (**AA**–**AZ**) Scatterplots of correlations between LPAR6 expression and gene markers of CD8+ T cell (**AA**,**AB**), general T cell (**AC**–**AE**), Treg (**AF**–**AI**), T cell exhaustion (**AJ**–**AN**), M2 macrophage (**AO**–**AQ**), monocyte (**AR**–**AS**) and dendritic cell (**AT**–**AZ**) in LUSC.

To study the association between LPAR6 and different types of TIICs, we focused on the correlations between the expression level of LPAR6 and immune marker sets of various immune cells of LUAD and LUSC. The correlations between LPAR6 expression level and immune marker gene sets of different immune cells, including CD8+ T cells, T cells (general), B cells, monocytes, TAMs, M1 and M2 macrophages, neutrophils, NK cells and DCs were determined in LUAD and LUSC (Table 2 and Figure 6). We also investigated the different types of T cells (Th1, Th2, Tfh, Th17, Tregs and exhausted T cells). After adjustment by purity, the correlation results revealed the LPAR6 expression level was significantly correlated with most immune marker sets of various immune cells and different subtypes of T cells, especially effect T cells in LUAD. However, none of these gene markers was significantly correlated with the LPAR6 expression level in LUSC and other cancer with poor prognosis (Table 2 and Figure 6).

**Figure 7:**
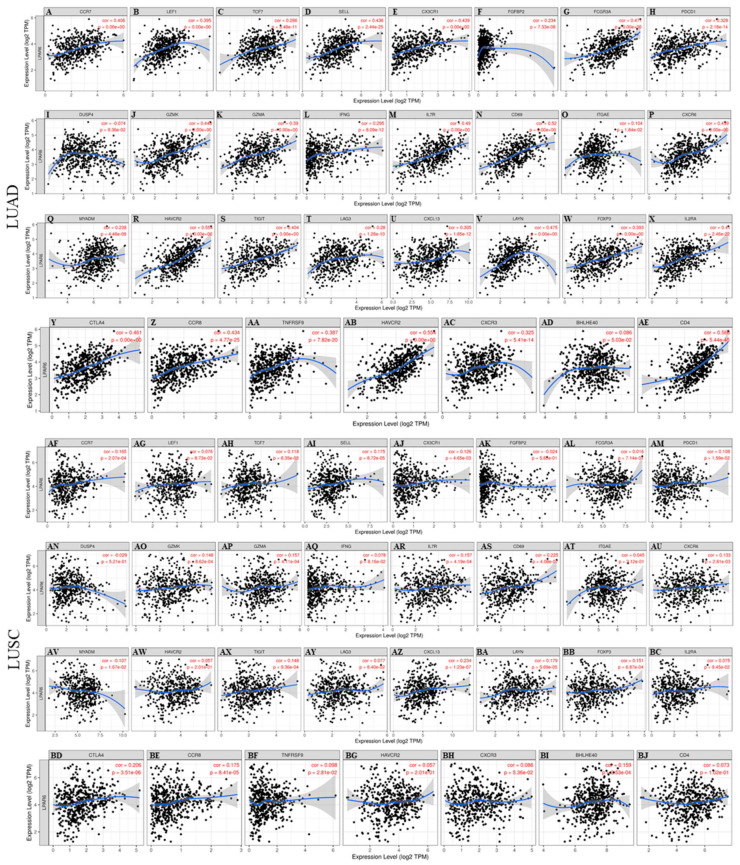
Correlation analysis between LPAR6 expression and various T cell marker sets in LUAD and LUSC. (**A**–**AE**) Scatterplots of correlations between LPAR6 expression and gene markers of Naive T-cell (CCR7, LEF1, TCF7, SELL) (**A**–**D**), Effector T-cell (CX3CR1, FGFBP2, FCGR3A) (**E**–**G**), Effector memory T-cell (PDCD1, DUSP4, GZMK, GZMA, IFNG) (**H**–**L**), Central memory T-cell (CCR7, SELL, IL7R) (**A**,**D**,**M**), Resident memory T-cell (CD69, ITGAE, CXCR6, MYADM) (**N**–**Q**), T cell exhaustion (HAVCR2, TIGIT, LAG3, PDCD1, CXCL13, LAYN) (**H**,**R**–**V**), Resting Treg (FOXP3, IL2RA) (**W**,**X**), Effector Treg (FOXP3, CTLA4, CCR8, TNFRSF9) (**W**,**Y**–**AA**), Th1-like (HAVCR2, IFNG, CXCR3, BHLHE40, CD4) (**L**,**AB**–**AE**) in LUAD; (**AF**–**BJ**) Scatterplots of correlations between LPAR6 expression and gene markers of Naive T-cell (CCR7, LEF1, TCF7, SELL) (**AF**–**AI**), Effector T-cell (CX3CR1, FGFBP2, FCGR3A) (**AJ**–**AL**), Effector memory T-cell (PDCD1, DUSP4, GZMK, GZMA, IFNG) (**AM**–**AQ**), Central memory T-cell (CCR7, SELL, IL7R) (**AF**,**AI**,**AR**), Resident memory T-cell (CD69, ITGAE, CXCR6, MYADM) (**AS**–**AV**), T cell exhaustion (HAVCR2, TIGIT, LAG3, PDCD1, CXCL13, LAYN) (**AM**,**AW**–**BA**), Resting Treg (FOXP3, IL2RA) (**BB**,**BC**), Effector Treg (FOXP3, CTLA4, CCR8, TNFRSF9) (**BB**,**BD**–**BF**), Th1-like (HAVCR2, IFNG, CXCR3, BHLHE40, CD4) (**AQ**,**BG**,**BH**, **BI**,**BJ**) in LUSC.

These results demonstrated that the mRNA expression levels of the marker genes in T cells (general, CD8+, Naive T, Effector T), natural killer (NK) cell, M1 macrophages and DCs have strong correlations with LPAR6 expression in LUAD (Table 2). More specifically, we demonstrated NOS2, IRF5, PTGS2 of M1 phenotype are significantly correlate with LPAR6 expression in LUAD (*p* < 0.0001; Figure 4A–H). It is reported that M1 could prevent tumor development. In-depth studies need to be done on whether LPAR6 is a crucial factor that mediating the de-polarization of macrophages and remodel tumor microenvironment. In addition, for Treg cells, LPAR6 does not demonstrate a correlation with the Tregs markers such as STAT5B in liver hepatocellular carcinoma (LIHC) (Table 2). Furthermore, we determined the association between the expression level of LPAR6 and the above marker sets of monocytes and various types of T cells in normal and tumor tissue in LUAD and LUSC (Supplementary Materials—Table S4, Figure 7).

### 3.8. Different Correlation Patterns between Tumor and Normal Tissue in LUAD Patients

**Figure 8:**
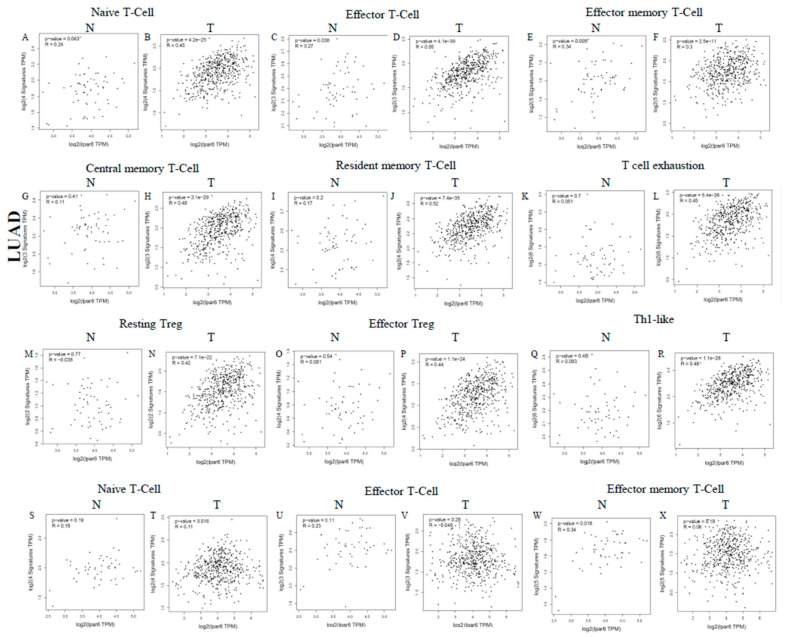

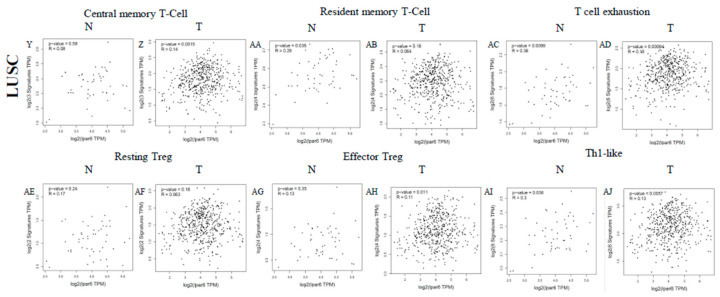
Correlation analysis between LPAR6 expression and various immune cells in normal and tumor tissue of LUAD and LUSC. (**A**–**R**) Scatterplots of correlations between LPAR6 expression and naive T-cell (**A**,**B**), effector T-cell (**C**,**D**), effector memory T-cell (**E**,**F**), central memory T-cell (**G**,**H**), resident memory T-cell (**I**,**J**), T cell exhaustion (**K**,**L**), resting Treg (**M**,**N**), effector Treg (**O**,**P**), Th1-like (**Q**,**R**) in the normal and tissue of LUAD; (**S**–**AJ**) Scatterplots of correlations between LPAR6 expression and gene markers of naive T-cell (**S**,**T**), effector T-cell (**U**,**V**), effector memory T-cell (**W**,**X**), central memory T-cell (**Y**,**Z**), resident memory T-cell (**AA**,**AB**), T cell exhaustion (**AC**,**AD**), resting Treg (**AE**,**AF**), effector Treg (**AG**,**AH**), Th1-like (**AI**,**AJ**) in LUSC.

The more interesting thing is that the expression levels of most of the marker sets of these immunocytes have strong correlations with LPAR6 expression in the tumor tissue of LUAD patients. In the LUSC, there was no significant correlation between LPAR6 and markers of immune cells (Figure 8, Supplementary Materials—Table S4). This finding suggests that there are different correlation patterns between tumor and normal tissue in LUAD patients. This exciting finding indicates that LPAR6 may regulate macrophage de-polarization in the tumor microenvironment of the LUAD and LPAR6 might be a novel target for LUAD therapy. High LPAR6 expression relates to a high infiltration level of DCs in the tumor tissue of LUAD patients, DC markers such as HLA-DQB1, CD1C and NRP1 show significant correlations with LPAR6 expression both in the tumor tissue in LUAD (Supplementary Materials—Table S4). These results further reveal that there is a strong relationship between LPAR6 and DCs infiltration.

### 3.9. Higher Expression Level of LPAR6 Was Correlated with Clinicopathological Parameters in LUAD Cohort and Was Correlated with Increased OS of LUAD and LUSC Patients

**Figure 9:**
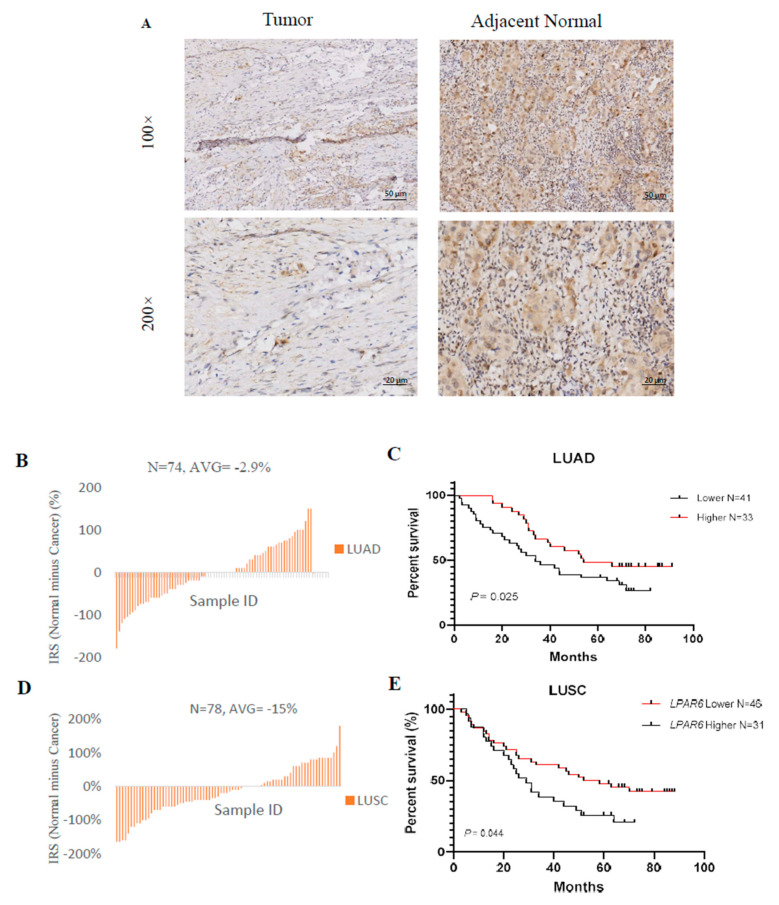

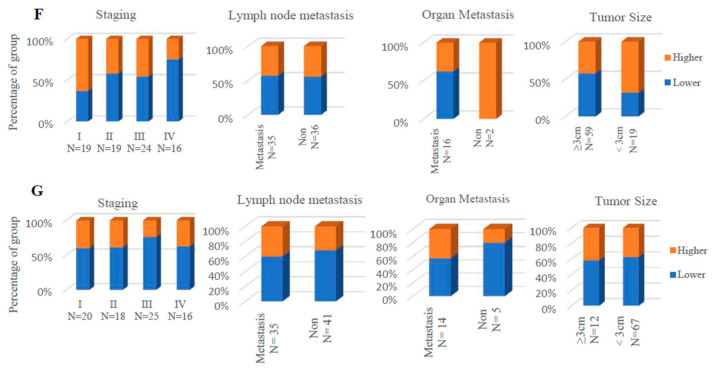
Higher expression of LPAR6 was correlated with clinicopathological parameters in LUAD cohort and was associated with increased overall survival (OS) of LUAD and LUSC patients. (**A**) Immunohistochemistry staining of the LPAR6 in the tumor and adjacent normal tissues. Red arrows indicated the cytoplasm-stained LPAR6. Bar, 50 μm; (**B**) The immunoreactive score (IRS) of the cytoplasm LPAR6 staining in 74 paired lung cancer tissues in LUAD cohort; (**C**) The Kaplan–Meier plot of the OS for lung cancer patients with relatively higher or lower LPAR6 expression levels in LUAD cohort (N = 74; Log-rank test, *p* = 0.02); (**D**) The IRS of the cytoplasm LPAR6 staining in 78 paired lung cancer tissues in LUSC cohort; (**E**) The Kaplan–Meier plot of the overall survival for lung cancer patients with relatively higher or lower LPAR6 expression levels in LUSC cohort (N = 78; log-rank test, *p* = 0.04). (**F**,**G**) The proportion of LPAR6 expression level (higher or lower) in different clinical stages, lymph node metastasis, organ metastasis and tumor size of patients in LUAD (**F**) and LUSC (**G**) cohorts.

We analyzed the protein level of LPAR6 in two independent lung cancer patient cohorts with 74 and 77 paired lung cancer and normal tissues, respectively. LPAR6 is mainly expressed in the cytoplasm of the cells (Figure 9A), and the protein level was lower in the lung cancer tissues compared with the normal tissues (Figure 9B,D). In the next step, we investigated the relationship between the LPAR6 protein level and the clinical characteristics of the LUAD and LUSC patient cohorts. Lung cancer patients with higher LPAR6 levels demonstrated better OS than those patients with relatively lower levels in LUAD patient cohorts, but not in LUSC patient cohorts (Figure 9C,E). Moreover, we found that lower LPAR6 was negatively correlated with the clinical stage of lung cancer and the lymph node metastasis of patients (Figure 9F,G).

In summary, we demonstrated that the LPAR6 was downregulated in the tumor tissue of LUAD patients and its mRNA expression was positive associated with the OS of LUAD patients base on the databases and TMA cohorts. The results further confirm that LPAR6 is specifically correlated with immune infiltrating cells in LUAD which suggests that LPAR6 plays a vital role in immune cells recruiting the tumor tissue in LUAD patients. LPAR6 and its modulation on tumor microenvironment may serve as a novel therapeutic target for LUAD.

## 4. Discussion

LPA receptors are G-protein-coupled receptor (GPCR) that bind to the LPA and trigger a series of down-streaming cellular responses, including cell proliferation, apoptosis and motility [48,49,50]. Previously, five LPA receptors (*LPAR*1-5) are well characterized and extensively studied [51]. LPAR6 is a recently determined GPCR, alias as ARWH1, HYPT8, LAH3, P2RY5, at first was considered as purinergic receptor P2Y5 that involved in inherited hair loss [23,52]. Although LPAR6 has not been extensively studied, it was reported that the LPAR6 suppresses tumor cell migration in colorectal cancer [28], and the expression of LPAR6 was decreased in P53-mutated cases [29]. It was also reported that the LPA axis plays a crucial role in hepatocellular carcinoma (HCC) by recruiting and trans-differentiating of peritumoral fibroblasts into TAMs [53]. This offers scientists a promising hint that LPAR6 is involved in the TME. Immunotherapy is a new genre of treatment for patients and has a tightly association with tumor microenvironment (TME) [29].

In this study, we announced that different mRNA expression levels of LPAR6 are associated with the prognostic potential in various types of cancer. Higher level of LPAR6 is associated with a better prognosis in three types of cancers, including liver cancer, lung cancer and breast cancer. Moreover, our data demonstrated that the immune-infiltration levels and diverse immune marker panels of the different subtypes of lung cancers (LUAD and LUSC) are associated with the expression level of LPAR6. To this end, our study provides a novel insight into elucidating the potential role of LPAR6 in tumor immunology and its usage as a prognostic biomarker and novel therapy target for LUAD.

In this work, we determined the LPAR6 expression levels and constructed a systematic prognostic landscape in various types of cancer using independent datasets and 33 type cancers of TCGA data in online database. The variation expression level of LPAR6 between cancer and normal tissues was determined in many cancer types. Relaying on the Oncomine database, we found that LPAR6, compared to normal tissues, was highly expressed in the tumor tissue of brain and CNS, kidney, gastric cancer, leukemia, lymphoma, liver and pancreatic cancer while some data sets showed that LPAR6 has a lower mRNA expression level in bladder, breast, cervical, colorectal, esophageal, lung and prostate cancer (Figure 1A). However, the redetermination of the TCGA data demonstrated that LPAR6 expression was more expressed in ESCA, KIRC, KIRP and THCA, but significantly lower expressed in BLCA, COAD, BRCA, HNSC, KICH, PRAD, LUAD, UCEC, READ and slightly lower in LIHC compared with paired adjacent normal tissues (Figure 1B). These vary in the mRNA expression levels of LPAR6 in different types of cancer among various databases might be a reflection in data collection approaches and underlying mechanisms involved in different biological properties. Nevertheless, we found similar prognostic associations between LPAR6 expression in bladder, breast, cervical, colorectal, esophageal, lung and prostate cancers in these databases. Investigation of the TCGA database enclosed that the higher LPAR6 expression level is correlated with better prognostic potential in ACC, LGG, skin cutaneous melanoma (SKCM). Furthermore, the determination of patient cohorts from PrognoScan database and Kaplan–Meier Plotter demonstrated a high mRNA expression level of LPAR6 is correlated with better prognosis in breast, lung, bladder, colorectal, eye and ovarian cancer (Figure 2). In two datasets of PrognoScan, higher LPAR6 expression levels could be considered as an independent risk factor for better prognosis in LUAD. Moreover, a high level of LPAR6 expression was shown to be correlated with a better prognosis of LUAD in the early stage with the lowest HR [0.27 (0.17–0.42)] for a better OS when LPAR6 was highly expressed in LUAD, rather than in LUSC. These together strongly suggest that LPAR6 could be a potential prognostic biomarker in LUAD.

Another crucial aspect is that the mRNA expression level of LPAR6 is correlated with diverse immune-infiltration levels in cancer, particularly in LUAD. Here, we demonstrate that there’s a strong positive correlation between the infiltration level of T cells (CD4+ T cells and CD8+ T cells), neutrophils, macrophages and DCs and LPAR6 expression in LUAD (Figure 3A,C). Moreover, the correlation patterns of the infiltration level are different in two types of lung cancers (LUSC and LUAD). The correlation between LPAR6 mRNA expression and the marker gene panel of immune cells implicates the role of LPAR6 in regulating tumor immunology in these types of cancer. A possible reason for this striking phenomenon might be that LPAR6 orchestrates the function of multiple immune marker gene sets. This supports the argument that the LPAR6 expression levels are important contributors to human malignancies and indicating the prognostic potential of specific types of cancer.

Firstly, the markers of M1 macrophages such as PTGS2 and IRF5 show a weak value correlation with LPAR6 expression in LUAD, respectively (Table 2). Since macrophages are functionally cells. M1 producing type 1 cytokines prevent tumors from developing, whereas type 2 and M2 macrophages M2 inducing type 2 cytokines facilitate tumor growth. Especially in the tumor tissue of LUAD, both IRF5 and NOS2 show significant correlations with LPAR6 expression and PTGS2 shows a significant correlation with LPAR6 expression in the tumor tissue (Supplementary Materials—Table S4). These results indicate the potential regulating role of LPAR6 in de-polarization macrophages against tumor that activated macrophages can be re-polarized into opposite functional phenotypes by microenvironmental modifications and then inhibit tumor growth.

Secondly, these results implied that LPAR6 has the potential to activate various types of T cells, including CD8+ T cell, naive T-cell, effector T-cell and natural killer cell and inactivate Tregs and decrease T cell exhaustion. CD8A, a crucial protein on T cells surface, is highly correlated with LPAR6 expression in LUAD which are types of cancers with better prognosis. Further, CD8A did not demonstrate a significant correlation pattern in LUSC (Table 2). This pattern also occurs with the general T cell markers such as CD3D, CD3E, CD2 and most markers of naive T-cell, effector memory T-cell, effector T-cell and natural killer cells, such as LEF1 which has been proven to be a predictor of better treatment response in AML. This is because a higher mRNA expression level of LEF1 was associated with favorable RFS in patients and predicted a significantly better overall survival for AML patients [54].

Thirdly, different correlation patterns have been found between the mRNA expression level of LPAR6 and the regulation of markers of T helper cells (Th1, Th2, Th17 and Tfh) in these different cancers. IFN-g is a Th1 cytokine with both pro- and anti-cancer properties [55] which are highly correlated with LPAR6 expression in LUAD, whereas it did not demonstrate significant correlations in LUSC (Table 2). IL-13 is an important immunoregulatory cytokine mainly produced by activated type II helper T cells and is widely involved in tumorigenesis and development, fibrosis and inflammation [56,57]. We found that IL-13 is highly correlated with *LPAR6* expression in LUAD, but did not demonstrate significant correlations in LUSC (adjusted by purity), and a similar situation is in the IL-21. So, these would be explanations that show why LPAR6 is an indication of poor prognosis in LUSC and a better prognosis in LUAD. We also studied the prognostic potential of LPAR6 in KIRC and the correlation of LPAR6 and immune-infiltration. We found that it is the same pattern with LUAD (data unshown). So, we believe that LPAR6 might also be a potential biomarker associated with KIRC also via immune infiltration.

All these correlations above are implying a potential mechanism that LPAR6 regulates T cell activities in LUAD. These findings indicate that the LPAR6 plays a crucial role in the recruitment and regulation of effective T cells infiltrating in LUAD patients would lead to a better prognosis.

## 5. Conclusions

In this study, we offered a potential explanation for the mechanism that why the mRNA expression level and protein of LPAR6 correlates with immune infiltration level and associates to a better prognostic potential in some specific types of cancer, especially in LUAD. Hence, the interactions between LPAR6 and the immunocytes in the tumor microenvironment could be an underlying mechanism for the correlation of LPAR6 expression level with the immune infiltration level and a better prognosis in LUAD patients.

## Figures and Tables

**Table 1:** Correlation of the mRNA expression level of *LPAR6* in different stage and clinical prognostic potential in lung Cancer with different clinicopathological factors.

Clinicopathological Characteristics	Overall Survival (*n* = 364)					
	LUAD (*n* = 720)			LUSC (*n* = 524)		
	N	Hazard Ratio	*p*-Value	N	Hazard Ratio	*p*-Value
Sex						
Female	318	0.39 (0.26–0.58)	**1.4 × 10^−10^**	129	1.69 (0.94–3.01)	0.075
Male	344	0.66 (0.48–0.93)	**0.015**	342	0.79 (0.59–1.04)	0.087
Smoking history						
Never	143	0.4 (0.17–0.96)	**0.034**	9	---	---
Smoker	246	0.49 (0.3–0.79)	**0.0029**	820	0.89 (0.72–1.09)	0.26
Stage						
1	370	0.27 (0.17–0.42)	**4.6 × 10^−10^**	172	0.75 (0.49–1.14)	0.17
2	136	0.51 (0.31–0.84)	**0.0073**	100	1.42 (0.76–2.65)	0.27
3	24	2.1 (0.71–6.21)	0.17	43	0.48 (0.24–0.96)	**0.035**
4	4	---	---	0	---	---

Bold values indicate *p* < 0.05.

**Table 2:** Correlation analysis between *LPAR6* and relate markers of immune cells.

Description	Gene Markers	LUAD				LUSC			
		None		Purity		None		Purity	
		Cor	*p*	Cor	*p*	Cor	*p*	Cor	*p*
CD8+ T cell	CD8A	0.363	***	0.238	***	0.121	*	0.065	0.157
	CD8B	0.366	***	0.281	***	0.231	***	0.196	***
T cell (general)	CD3D	0.451	***	0.318	***	0.197	***	0.136	*
	CD3E	0.434	***	0.283	***	0.163	**	0.092	0.045
	CD2	0.49	***	0.358	***	0.171	**	0.101	0.0277
Naive T-Cell	CCR7	0.39	***	0.222	***	0.175	***	0.109	0.0175
	LEF1	0.351	***	0.241	***	−0.002	0.962	0.014	0.766
	TCF7	0.237	***	0.102	0.0232	0.095	0.0336	0.049	0.289
	SELL	0.421	***	0.26	***	0.187	***	0.115	0.12
Effector T-Cell	CX3CR1	0.41	***	0.353	***	0.113	0.0113	0.055	0.23
	FGFBP2	0.247	***	0.185	***	−0.04	0.378	−0.02	0.656
	FCGR3A	0.448	***	0.354	***	0.042	0.35	−0.044	0.34
Effector memory T-Cell	PDCD1	0.316	***	0.175	***	0.112	0.0121	0.045	0.323
	DUSP4	−0.074	0.0915	−0.075	0.0966	−0.017	0.707	−0.057	0.212
	GZMK	0.444	***	0.309	***	0.172	**	0.106	0.0204
	GZMA	0.408	***	0.295	***	0.197	***	0.143	*
	IFNG	0.304	***	0.201	***	0.101	0.0235	0.061	0.183
Resident memory T-Cell	CD69	0.518	***	0.423	***	0.247	***	0.192	***
	ITGAE	0.295	***	0.228	***	0.109	0.0149	0.09	0.0493
	CXCR6	0.434	***	0.305	***	0.161	**	0.095	0.0389
	MYADM	0.162	**	0.064	0.156	−0.132	*	−0.197	***
B cell	CD19	0.341	***	0.192	***	0.157	**	0.083	0.0694
	CD79A	0.312	***	0.171	**	0.155	**	0.076	0.096
Monocyte	CD86	0.55	***	0.455	***	0.164	**	0.079	0.085
	CD115 (CSF1R)	0.495	***	0.388	***	0.071	0.111	−0.031	0.498
TAM	CCL2	0.424	***	0.331	***	0.148	**	0.086	0.0611
	CD68	0.387	***	0.291	***	0.012	0.785	−0.087	0.0576
	IL10	0.523	***	0.433	***	0.211	***	0.151	**
M1 Macrophage	INOS (NOS2)	0.14	*	0.075	0.0955	0.104	0.0203	0.106	0.0203
	IRF5	0346	***	0.254	***	−0.101	0.0236	−0.129	**
	COX2 (PTGS2)	0.009	0.833	0.017	0.705	0.27	***	0.244	***
M2 Macrophage	CD163	0.376	***	0.281	***	0.032	0.472	−0.058	0.209
	VSIG4	0.438	***	0.358	***	0.061	0.171	−0.021	0.649
	MS4A4A	0.501	***	0.412	***	0.109	0.0145	0.028	0.536
Neutrophils	CD66b (CEACAM8)	0.114	*	0.09	0.0464	0.023	0.613	0.006	0.894
	CD11b (ITGAM)	0.405	***	0.29	***	0.091	0.0412	−0.006	0.898
	CCR7	0.39	***	0.222	***	0.175	***	0.109	0.0175
Natural killer cell	KIR2DL1	0.117	*	0.064	0.155	0.081	0.0704	0.052	0.258
	KIR2DL3	0.209	***	0.13	*	0.013	0.776	0.013	0.776
	KIR2DL4	0.179	***	0.11	0.0145	0.08	0.0744	0.039	0.4
	KIR3DL1	0.149	**	0.075	0.098	0.005	0.902	−0.048	0.294
	KIR3DL2	0.168	**	0.078	0.083	0.015	0.737	−0.038	0.41
	KIR3DL3	0.039	0.38	0.006	0.899	−0.116	*	−0.142	*
	KIR2DS4	0.143	*	0.065	0.149	0.052	0.245	0.026	0.572
Dendritic cell	HLA-DPB1	0.463	***	0.353	***	0.098	0.0279	0.014	0.759
	HLA-DQB1	0.315	***	0.195	***	0.098	0.0279	0.014	0.759
	HLA-DRA	0.476	***	0.376	***	0.135	*	0.062	0.178
	HLA-DPA1	0.447	***	0.343	***	0.106	0.0172	0.029	0.521
	BDCA-1 (CD1C)	0.382	***	0.294	***	0.196	***	0.131	*
	BDCA-4 (NRP1)	0.18	***	0.137	*	0.052	0.247	−0.014	0.767
	CD11c (ITGAX)	0.474	***	0.364	***	0.168	**	0.082	0.074
Th1	TBX21 (T-bet)	0.355	***	0.216	***	0.119	*	0.051	0.266
	STAT4	0.394	***	0.267	***	0.195	***	0.122	*
	STAT1	0.193	***	0.075	0.094	0.032	0.477	−0.018	0.689
	IFNG (IFN-g)	0.304	***	0.201	***	0.101	0.0235	0.061	0.183
	TNF-a (TNF)	0.414	***	0.291	***	0.277	***	0.229	***
Th2	GATA3	0.365	***	0.231	***	0.187	***	0.143	*
	STAT6	0.007	0.873	0.019	0.681	0.257	***	0.259	***
	STAT5A	0.459	***	0.33	***	0.15	**	0.082	0.0722
	IL13	0.209	***	0.128	0.0213	0.076	0.0818	0.037	0.423
Tfh	BCL6	0.022	0.612	0.018	0.684	0.08	0.0729	0.105	0.0223
	IL21	0.118	**	0.038	0.402	0.034	0.452	−0.013	0.782
Th17	STAT3	−0.138	**	−0.147	**	0.129	*	0.109	0.0168
	IL17A	0.177	***	0.11	0.014	0.051	0.254	0.025	0.58
Treg	FOXP3	0.364	***	0.207	***	0.145	*	0.063	0.172
	CCR8	0.382	***	0.242	***	0.162	**	0.083	0.0706
	STAT5B	0.212	***	0.18	***	−0.071	0.114	−0.076	0.096
	TGFB1 (TGFb)	0.304	***	0.201	***	0.123	**	0.123	***
T cell exhaustion	PDCD1 (PD-1)	0.316	***	0.175	***	0.112	0.0121	0.045	0.323
	CTLA4	0.444	***	0.304	***	0.217	***	0.152	**
	LAG3	0.275	***	0.152	***	0.095	0.0329	0.034	0.457
	HAVCR2 (TIM-3)	0.539	***	0.442	***	0.094	0.0357	0.008	0.867
	GZMB	0.268	***	0.15	***	0.137	**	0.077	0.0926

TAM, tumor-associated macrophage; Th, T helper cell; Tfh, Follicular helper T cell; Treg, regulatory T cell; Cor, R value of Spearman’s correlation; None, correlation without adjustment. Purity, correlation adjusted by purity. * *p* < 0.01; ** *p* < 0.001; *** *p* < 0.0001.

## Data Availability

The data analyzed or generated during this study are included in this article and its supplementary information.

## Supplementary Materials

The following are available online at https://www.mdpi.com/article/10.3390/ijerph182111038/s1, Figure S1: Kaplan-Meier survival curves comparing the high and low expression of LPAR6 in different types of cancer in the PrognoScan (A–K) and Kaplan–Meier plotter databases (L–Q). (A) Survival curves of DSS in bladder cancer cohort [GSE13507 (n = 165, *p* = 0.0067285)]. (B–D) Survival curves of OS, RFS and DFS in the breast cancer cohort [GSE1456-GPL96 (n = 159, *p* = 0.00575883; *p* = 0.0000252; *p* = 0.000210173)]. (E-G) Survival curves of DMFS in the breast cancer cohort [GSE19615 (n = 159, *p* = 0.00575883), GSE9195 (n = 159, *p* = 0.0466683), GSE11121 (n = 200, *p* = 0.0389008)]. (H-J) Survival curves of DSS in the breast, DFS in the colorectal and DMFS in the Eye Cancer cohort [GSE3494 (n = 236, *p* = 0.00294205), GSE17537 (n = 55, *p* = 0.0257972), GSE22138 (n = 63, *p* = 0.00092478)]. (K) Survival curves of PFS in the ovarian cancer cohort [GSE17260 (n = 110, *p* = 0.0392865)]. (L-N) Survival curves of OS (n = 1402), RFS (n = 3951) and DMSF (n = 1746) in the breast cancer cohorts. (O-Q) Survival curves of OS (n = 364), FPS (n = 370) and RFS (n = 316) in the liver cancer cohort. Supplementary Figure S2: Promoter methylation levels of LPAR6 impacts the clinicopathological parameters in LUAD and LUSC cohorts. Supplementary Figure S3: The correlation of the expression of LPAR6 with immune infiltration level in cancers. Supplementary Material Table S1. LPAR6 expression in Oncomine database; Supplementary Material Table S2. Relation between LPAR6 expression and patient prognosis of different cancer in Prognoscan database; Supplementary Material Table S3: The expression level of LPAR6 is correlated with immune infiltration level in various types of cancers; Supplementary Material Table S4: Correlation analysis between LPAR6 and relate genes and markers of immune cells in tumor and normal tissue in LUAD and LUSC.
