# Supplementary material for: Lysophosphatidic Acid Receptor 6 (LPAR6) Is a Potential Biomarker Associated with Lung Adenocarcinoma
Source: Int J Environ Res Public Health. 2021 Oct 20;18(21):11038. doi: 10.3390/ijerph182111038 (PMC8583018; doi:10.3390/ijerph182111038)
Supplement: Supplementary file 1 [file ijerph-18-11038-s001.zip › ijerph-1339832-supplementary/SI-Fig S1_R2.pptx]

## Slide 1
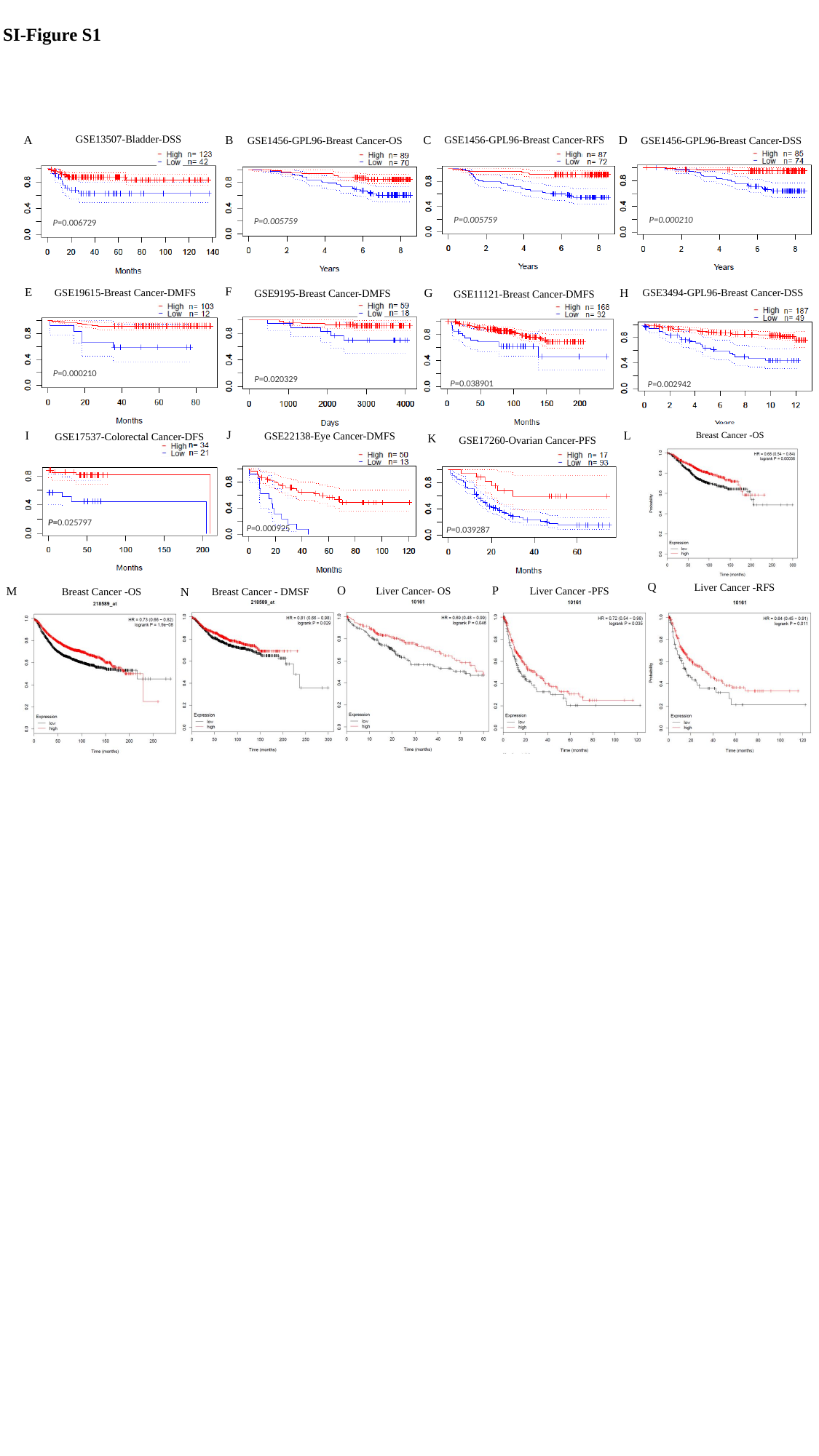

SI-Figure S1
A
B
D
C
GSE13507-Bladder-DSS
GSE1456-GPL96-Breast Cancer-RFS
GSE1456-GPL96-Breast Cancer-OS
GSE1456-GPL96-Breast Cancer-DSS
Probability
| P=0.005759 |
| --- |
| P=0.000210 |
| --- |
| P=0.005759 |
| --- |
| P=0.006729 |
| --- |
F
E
G
H
GSE3494-GPL96-Breast Cancer-DSS
GSE19615-Breast Cancer-DMFS
GSE9195-Breast Cancer-DMFS
GSE11121-Breast Cancer-DMFS
Probability
| P=0.000210 |
| --- |
P=0.020329
P=0.038901
P=0.002942
J
I
L
GSE22138-Eye Cancer-DMFS
Breast Cancer -OS
GSE17537-Colorectal Cancer-DFS
K
GSE17260-Ovarian Cancer-PFS
Probability
| P=0.025797 |
| --- |
P=0.000925
P=0.039287
Q
Liver Cancer -RFS
P
O
M
Liver Cancer -PFS
Liver Cancer- OS
N
Breast Cancer - DMSF
Breast Cancer -OS
Probability
