# Supplementary material for: Lysophosphatidic Acid Receptor 6 (LPAR6) Is a Potential Biomarker Associated with Lung Adenocarcinoma
Source: Int J Environ Res Public Health. 2021 Oct 20;18(21):11038. doi: 10.3390/ijerph182111038 (PMC8583018; doi:10.3390/ijerph182111038)
Supplement: Supplementary file 1 [file ijerph-18-11038-s001.zip › ijerph-1339832-supplementary/SI-Fig S2_R2new.pptx]

## Slide 1
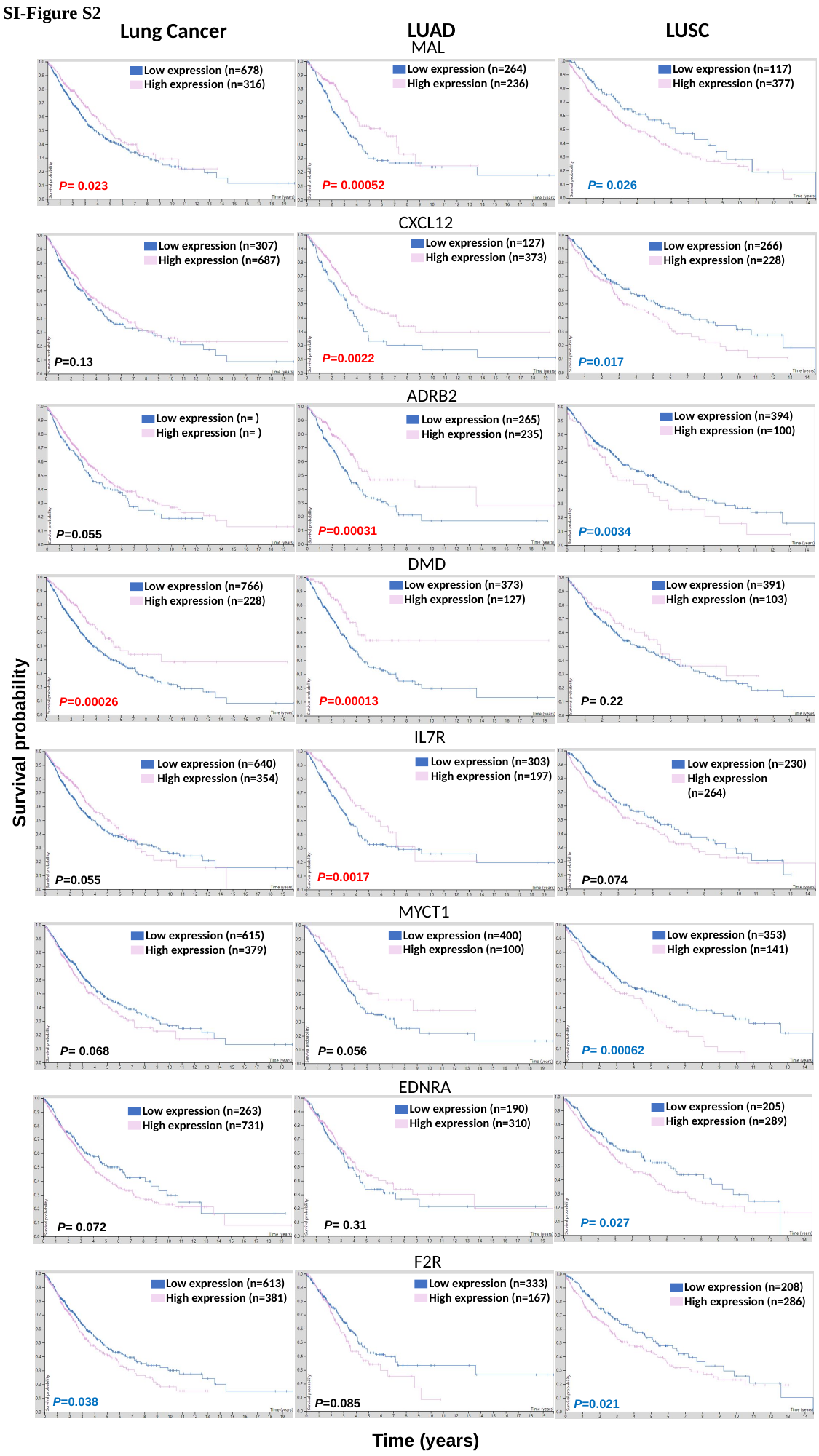

SI-Figure S2
LUAD
LUSC
Lung Cancer
MAL
Low expression (n=264)
High expression (n=236)
P= 0.00052
Low expression (n=117)
High expression (n=377)
P= 0.026
Low expression (n=678)
High expression (n=316)
P= 0.023
CXCL12
Low expression (n=127)
High expression (n=373)
Low expression (n=307)
High expression (n=687)
Low expression (n=266)
High expression (n=228)
P=0.0022
P=0.13
P=0.017
ADRB2
Low expression (n=394)
High expression (n=100)
Low expression (n=265)
High expression (n=235)
P=0.00031
P=0.0034
Low expression (n= )
High expression (n= )
P=0.055
DMD
Low expression (n=373)
High expression (n=127)
P=0.00013
Low expression (n=391)
High expression (n=103)
P= 0.22
Low expression (n=766)
High expression (n=228)
P=0.00026
IL7R
Low expression (n=303)
High expression (n=197)
Low expression (n=230)
High expression (n=264)
Low expression (n=640)
High expression (n=354)
P=0.055
P=0.0017
P=0.074
Survival probability
MYCT1
Low expression (n=353)
High expression (n=141)
P= 0.00062
Low expression (n=400)
High expression (n=100)
P= 0.056
Low expression (n=615)
High expression (n=379)
P= 0.068
EDNRA
Low expression (n=205)
High expression (n=289)
P= 0.027
Low expression (n=190)
High expression (n=310)
P= 0.31
Low expression (n=263)
High expression (n=731)
P= 0.072
F2R
Low expression (n=333)
High expression (n=167)
P=0.085
Low expression (n=613)
High expression (n=381)
Low expression (n=208)
High expression (n=286)
P=0.021
P=0.038
Time (years)
