# Supplementary material for: Lysophosphatidic Acid Receptor 6 (LPAR6) Is a Potential Biomarker Associated with Lung Adenocarcinoma
Source: Int J Environ Res Public Health. 2021 Oct 20;18(21):11038. doi: 10.3390/ijerph182111038 (PMC8583018; doi:10.3390/ijerph182111038)
Supplement: Supplementary file 1 [file ijerph-18-11038-s001.zip › ijerph-1339832-supplementary/SI-Table S1_R2.pdf]

## Supplementary Table S1. *LPAR6* expression in Oncomine database

| Cancer | Cancer type (Tumor vs Normal) | *P-value* | Fold change | Rank (%) | Sample | PubMed ID |
|---|---|---|---|---|---|---|
| Bladder Cancer | Infiltrating Bladder Urothelial Carcinoma | 2.84E-7 | -1.695 | 1 | 93 | 15930339 |
| | Infiltrating Bladder Urothelial Carcinoma | 2.11E-9 | -2.136 | 2 | 256 | 20421545 |
| Brain and CNS Cancer | Glioblastoma | 4.37E-6 | 2.526 | 5 | 54 | 16204036 |
| Breast Cancer | Invasive Breast Carcinoma | 7.04E-26 | -1.968 | 1 | 158 | 21373875 |
| | Invasive Ductal Breast Carcinoma Stroma | 2.14E-5 | -2.328 | 1 | 66 | 19187537 |
| | Ductal Breast Carcinoma in Situ Epithelia | 3.17E-5 | -3.812 | 2 | 66 | 19187537 |
| | Invasive Ductal Breast Carcinoma | 7.39E-9 | -1.957 | 1 | 64 | 15034139 |
| | Invasive Breast Carcinoma | 2.11E-21 | -2.376 | 3 | 593 | Not Published |
| | Invasive Ductal Breast Carcinoma | 1.00E-31 | -3.316 | 4 | 593 | Not Published |
| | Mixed Lobular and Ductal Breast Carcinoma | 1.21E-5 | -1.961 | 4 | 593 | Not Published |
| | Invasive Lobular Breast Carcinoma | 1.16E-11 | -2.104 | 5 | 593 | Not Published |
| | Ductal Breast Carcinoma | 1.06E-7 | -3.021 | 3 | 47 | 16473279 |
| Cervical Cancer | Cervical Squamous Cell Carcinoma | 6.37E-10 | -4.015 | 1 | 66 | 18506748 |
| | Cervical Squamous Cell Carcinoma Epithelia | 1.43E-5 | -2.374 | 3 | 41 | 17974957 |
| Colorectal Cancer | Colon Adenocarcinoma | 3.46E-8 | -1.820 | 4 | 123 | 17640062 |
| Esophageal Cancer | Esophageal Adenocarcinoma | 4.42E-22 | -3.415 | 2 | 118 | 21152079 |
| | Barrett's Esophagus | 3.84E-11 | -3.801 | 4 | 118 | 21152079 |
| Gastric Cancer | Gastric Mixed Adenocarcinoma | 7.56E-5 | 2.090 | 4 | 69 | 19081245 |
| Kidney Cancer | Clear Cell Renal Cell Carcinoma | 2.45E-7 | 2.852 | 3 | 20 | 17699851 |
| | Hereditary Clear Cell Renal Cell Carcinoma | 9.28E-9 | 2.194 | 5 | 70 | 19470766 |
| Leukemia | T-Cell Childhood Acute Lymphoblastic Leukemia | 6.41E-15 | 16.279 | 1 | 288 | 21487112 |
| | B-Cell Childhood Acute Lymphoblastic Leukemia | 4.81E-6 | 7.365 | 1 | 288 | 21487112 |
| | B-Cell Childhood Acute Lymphoblastic Leukemia | 1.99E-63 | 4.774 | 2 | 2096 | 20406941 |
| | T-Cell Acute Lymphoblastic Leukemia | 1.65E-31 | 4.050 | 4 | 2096 | 20406941 |
| | B-Cell Acute Lymphoblastic Leukemia | 5.25E-27 | 3.157 | 5 | 2096 | 20406941 |
| | B-Cell Acute Lymphoblastic Leukemia | 7.52E-5 | 7.367 | 2 | 28 | 16267031 |
| | T-Cell Prolymphocytic Leukemia | 6.71E-5 | -5.486 | 1 | 14 | 17713554 |
| | Acute Adult T-Cell Leukemia/Lymphoma | 1.04E-5 | -7.409 | 1 | 47 | 16909099 |
| Liver cancer | Hepatocellular Carcinoma | 1.52E-12 | 2.552 | 1 | 115 | 19098997 |
| | Cirrhosis | 2.51E-15 | 2.976 | 3 | 115 | 19098997 |
| Lymphoma | Follicular Lymphoma | 5.58E-24 | 5.016 | 2 | 136 | 19412164 |
| | Diffuse Large B-Cell Lymphoma | 9.76E-19 | 3.635 | 3 | 136 | 19412164 |
| | Acute Adult T-Cell Leukemia/Lymphoma | 1.04E-5 | -7.409 | 1 | 47 | 16909099 |
| | Classical Hodgkin's Lymphoma | 6.66E-6 | -1.810 | 2 | 64 | 19657361 |
| Pancreatic Cancer | Pancreatic Adenocarcinoma | 4.95E-5 | 2.038 | 2 | 36 | 12651607 |
| Prostate Cancer | Prostate Adenocarcinoma | 9.89E-6 | -1.542 | 2 | 89 | 18245496 |
| Other | Teratoma, NOS | 7.22E-8 | 3.869 | 4 | 107 | 16424014 |
