# Supplementary material for: Lysophosphatidic Acid Receptor 6 (LPAR6) Is a Potential Biomarker Associated with Lung Adenocarcinoma
Source: Int J Environ Res Public Health. 2021 Oct 20;18(21):11038. doi: 10.3390/ijerph182111038 (PMC8583018; doi:10.3390/ijerph182111038)
Supplement: Supplementary file 1 [file ijerph-18-11038-s001.zip › ijerph-1339832-supplementary/SI-Table S3_R2.pdf]

Supplementary-Table S3 | The expression level of LPAR6 is correlated with immune infiltration level in various types of cancers

Relationship		Cancer Type
Negative correlations	Tumor purity (26)	BLCA, BRCA, BRCA-Basal, BRCA-Her2, BRCA-Luminal, CESC, CHOL, DLBC, GBM, HNSC, HNSC-HPVneg, KICH, KIRC, KIRP, LGG, LIHC, LUAD, LUSC, OV, PCPG, SARC, SKCM, SKCM-Metastasis, STAD, TGCT, UCEC, UVM
Positive correlations	B cells (13)	BRCA-Basal, BRCA-Luminal, CHOL, COAD, GBM, HNSC-HPVpos, KIRC, LGG, LIHC, LUAD, THCA, THYM, UCEC
	CD8+ T cells (24)	ACC, BRCA, BRCA-Basal, BRCA-Her2, BRCA-Luminal, CESC, COAD, HNSC, HNSC-HPVpos, HNSC-HPVneg, KIRC, KIRP, LGG, LIHC, LUAD, MESO, OV, READ, SKCM, SKCM-Primary, SKCM-Metastasis, STAD, THCA, UCEC
	CD4+ T cells (26)	ACC, BLCA, BRCA, BRCA-Basal, BRCA-Her2, BRCA-Luminal, CESC, CHOL, COAD, DLBC, KICH, KIRC, KIRP, LGG, LIHC, LUAD, MESO, OV, PAAD, PCPG, STAD, TGCT, UCEC, USC
	Macrophages (20)	ACC, BRCA, BRCA-Basal, BRCA-Her2, BRCA-Luminal, CHOL, COAD, HNSC, HNSC-HPVneg, KIRP, LGG, LIHC, LUAD, MESO, OV, PCPG, SKCM, SKCM-Metastasis, STAD, UCEC
	Neutrophils (33)	ACC, BLCA, BRCA, BRCA-Basal, BRCA-Her2, BRCA-Luminal, CESC, COAD, ESCA, HNSC, HNSC-HPVpos, HNSC-HPVneg, KIRC, KIRP, LGG, LIHC, LUAD, LUSC, OV, PAAD, READ, SKCM, SKCM-Primary, SKCM-Metastasis, STAD, TGCT, THCA, THYM, UCEC, UCS, UVM
	Dendritic cells (21)	ACC, BRCA, BRCA-Basal, BRCA-Her2, BRCA-Luminal, CESC, CHOL, COAD, ESCA, KIRC, KIRP, LGG, LIHC, LUAD, OV, PCPG, SKCM, STAD, THCA, THYM, UCEC
