# Supplementary material for: Lysophosphatidic Acid Receptor 6 (LPAR6) Is a Potential Biomarker Associated with Lung Adenocarcinoma
Source: Int J Environ Res Public Health. 2021 Oct 20;18(21):11038. doi: 10.3390/ijerph182111038 (PMC8583018; doi:10.3390/ijerph182111038)
Supplement: Supplementary file 1 [file ijerph-18-11038-s001.zip › ijerph-1339832-supplementary/SI-Table S4_R2.pdf]

Supplementary-Table S4 | Correlation analysis between LPAR6 and relate genes and markers of immune cells in tumor and normal tissue in LUAD and LUSC

Description	Gene markers	LUAD				LUSC			
		Tumor		Normal		Tumor		Normal	
		R	P	R	P	R	P	R	P
**CD8+ T cell**	CD8A	0.34	***	0.15	0.24	0.047	0.3	0.36	*
	CD8B	0.33	***	0.12	0.38	0.16	***	0.36	0.011
**T cell (general)**	CD3D	0.41	***	0.26	0.05	0.11	0.014	0.35	0.014
	CD3E	0.41	***	0.2	0.13	0.083	0.067	0.19	0.18
	CD2	0.46	***	0.26	0.05	0.094	0.039	0.34	0.017
**Naive T-Cell**	CCR7	0.40	***	0.06	0.65	0.11	0.014	0.11	0.45
	LEF1	0.37	***	0.43	**	0.042	0.36	0.39	*
	TCF7	0.27	***	0.29	0.025	0.06	0.18	0.25	0.081
	SELL	0.42	***	0.052	0.7	0.12	*	0.07	0.63
**Effector T-Cell**	CX3CR1	0.44	***	0.38	*	0.11	0.02	0.31	0.028
	FGFBP2	0.23	***	0.2	0.13	−0.023	0.61	0.18	0.21
	FCGR3A	0.46	***	-0.16	0.23	−0.026	0.57	0.01	0.95
**Effector memory T-Cell**	PDCD1	0.31	***	-0.16	0.22	0.05	0.27	0.026	0.86
	DUSP4	−0.086	0.06	0.16	0.23	−0.05	0.27	0.16	0.26
	GZMK	0.42	***	0.3	0.021	0.093	0.04	0.32	0.021
	GZMA	0.36	***	0.41	*	0.12	*	0.47	**
	IFNG	0.28	***	0.35	*	0.048	0.29	0.39	*
**Central memory T-Cell**	CCR7	0.4	***	0.06	0.65	0.11	0.014	0.11	0.45
	SELL	0.42	***	0.052	0.7	0.12	*	0.069	0.63
	IL7R	0.47	***	0.24	0.063	0.13	*	0.032	0.82
**Resident memory T-Cell**	CD69	0.5	***	0.37	*	0.18	***	0.49	**
	ITGAE	0.098	0.032	0.27	-0.15	0.059	0.19	0.29	0.04
	CXCR6	0.41	***	0.18	0.17	0.086	0.058	0.053	0.71
	MYADM	0.24	***	-0.1	0.44	−0.13	*	−0.11	0.43
**B cell**	CD19	0.29	***	0.061	0.65	0.053	0.25	0.22	0.12
	CD79A	0.23	***	−0.0023	0.99	0.051	0.26	0.13	0.37
**Monocyte**	CD86	0.57	***	−0.04	0.76	0.1	0.023	0.12	0.39
	CD115 (CSF1R)	0.54	***	−0.029	0.82	0.041	0.36	0.12	0.4
**TAM**	CCL2	0.44	***	0.18	0.17	0.12	*	0.14	0.35
	CD68	0.37	***	−0.11	0.42	−0.036	0.42	−0.22	0.13
	IL10	0.53	***	0.099	0.46	0.17	**	0.49	**
**M1 Macrophage**	INOS (NOS2)	0.19	***	0.19	0.15	0.12	*	0.014	0.92
	IRF5	0.34	***	−0.19	0.16	−0.094	0.039	0.035	0.81
	COX2 (PTGS2)	0.066	0.15	0.0054	0.97	0.27	***	0.083	0.57
**M2 Macrophage**	CD163	0.44	***	−0.33	0.012	0.014	0.76	−0.14	0.32
	VSIG4	0.46	***	−0.28	0.03	0.99	***	−0.18	0.22
	MS4A4A	0.52	***	−0.15	0.25	0.04	0.38	0.12	0.42
**Neutrophils**	CD66b (CEACAM8)	0.12	*	0.027	0.84	0.028	0.54	−0.15	0.3
	CD11b (ITGAM)	0.47	***	−0.16	0.23	0.083	0.068	0.071	0.62
	CCR7	0.4	***	0.06	0.65	0.11	0.014	0.11	0.45
**Natural killer cell**	KIR2DL1	0.13	*	0.14	0.28	0.022	0.63	0.18	0.21
	KIR2DL3	0.2	***	0.37	*	0.014	0.75	0.29	0.041

	KIR2DL4	0.16	**	0.13	0.32	0.015	0.74	0.27	0.06
	KIR3DL1	0.18	**	0.19	0.15	−0.015	0.74	0.25	0.079
	KIR3DL2	0.17	**	0.26	0.048	−0.02	0.65	0.17	0.24
	KIR3DL3	0.0028	0.95	0.2	0.13	−0.17	**	−0.12	0.41
	KIR2DS4	0.12	*	0.32	0.014	0.026	0.56	0.23	0.11
**Dendritic cell**	HLA-DPB1	0.45	***	−0.11	0.41	0.022	0.62	−0.15	0.29
	HLA-DQB1	0.23	***	0.025	0.85	0.031	0.49	−0.25	0.075
	HLA-DRA	0.46	***	−0.086	0.52	0.059	0.19	0.046	0.75
	HLA-DPA1	0.43	***	−0.072	0.59	0.031	0.5	−0.12	0.41
	BDCA-1(CD1C)	0.37	***	0.3	0.019	0.15	*	0.4	*
	BDCA-4(NRP1)	0.21	***	0.47	**	0.045	0.32	0.25	0.084
	CD11c (ITGAX)	0.5	***	−0.21	0.11	0.13	*	−0.069	0.63
**Th1**	TBX21 (T-bet)	0.36	***	0.12	0.35	0.062	0.17	0.26	0.074
	STAT4	0.39	***	0.069	0.6	0.16	**	0.33	0.021
	STAT1	0.24	***	0.32	0.014	−0.0066	0.88	0.22	0.13
	IFNG (IFN-g)	0.28	***	0.35	*	0.048	0.29	0.39	0.005
	TNF-a (TNF)	0.43	***	0.23	0.086	0.25	***	0.19	0.19
**Th2**	GATA3	0.37	***	0.21	0.12	0.18	***	0.32	0.022
	STAT6	0.13	*	0.15	0.26	0.25	***	0.19	0.18
	STAT5A	0.47	***	0.063	0.64	0.11	0.017	−0.029	0.84
	IL13	0.26	***	0.29	0.027	0.092	0.043	0.047	0.74
**Tfh**	BCL6	0.13	*	−0.13	0.33	0.1	0.026	−0.22	0.12
	IL21	0.21	***	0.11	0.39	−0.0057	0.9	0.057	0.7
**Th17**	STAT3	0.041	0.37	−0.14	0.29	0.14	*	−0.095	0.51
	IL17A	0.17	**	0.01	0.94	−0.0034	0.94	0.029	0.84
**Treg**	FOXP3	0.37	***	0.069	0.61	0.097	0.032	0.13	0.36
	CCR8	0.41	***	0.13	0.33	0.13	*	0.15	0.29
	STAT5B	0.28	***	0.33	0.01	−0.021	0.64	0.35	0.013
	TGFB1 (TGFb)	0.32	***	0.065	0.62	0.061	0.18	0.086	0.55
**T cell exhaustion**	PDCD1 (PD-1)	0.31	***	−0.16	0.22	0.05	0.27	0.026	0.86
	CTLA4	0.45	***	0.15	0.24	0.14	*	0.21	0.15
	LAG3	0.26	***	0.046	0.73	0.037	0.41	0.31	0.028
	HAVCR2 (TIM-3)	0.55	***	−0.15	0.25	0.02	0.67	0.073	0.61
	GZMB	0.22	***	0.14	0.3	0.063	0.16	0.27	0.055
	TIGIT	0.38	***	0.15	0.27	0.096	0.034	0.36	*
	CXCL13	0.28	***	−0.09	0.5	0.19	***	0.18	0.22
	LAYN	0.47	***	0.32	0.012	0.18	***	0.59	***
**Effector Treg**	FOXP3	0.37	***	0.069	0.61	0.097	0.032	0.13	0.36
	CTLA4	0.45	***	0.15	0.24	0.14	*	0.21	0.15
	CCR8	0.41	***	0.13	0.33	0.13	*	0.15	0.29
	TNFRSF9	0.35	***	−0.056	0.67	0.067	0.14	0.0067	0.96
**Th1-like**	CXCL13	0.28	***	−0.09	0.5	0.19	***	0.18	0.22
	HAVCR2	0.55	***	−0.15	0.25	0.02	0.67	0.073	0.61
	IFNG	0.31	***	0.35	*	0.048	0.29	0.39	*
	CXCR3	0.3	***	0.19	0.14	0.027	0.55	0.26	0.073
	BHLHE40	0.086	0.06	−0.073	0.58	0.16	**	0.041	0.78
	CD4	0.55	***	0.23	0.081	0.025	0.58	0.2	0.16

*$P < 0.01$; **$P < 0.001$; ***$P < 0.0001$.
