# Supplementary figures and images for: Lysophosphatidic Acid Receptor 6 (LPAR6) Is a Potential Biomarker Associated with Lung Adenocarcinoma
Source: Int J Environ Res Public Health. 2021 Oct 20;18(21):11038. doi: 10.3390/ijerph182111038 (PMC8583018; doi:10.3390/ijerph182111038)

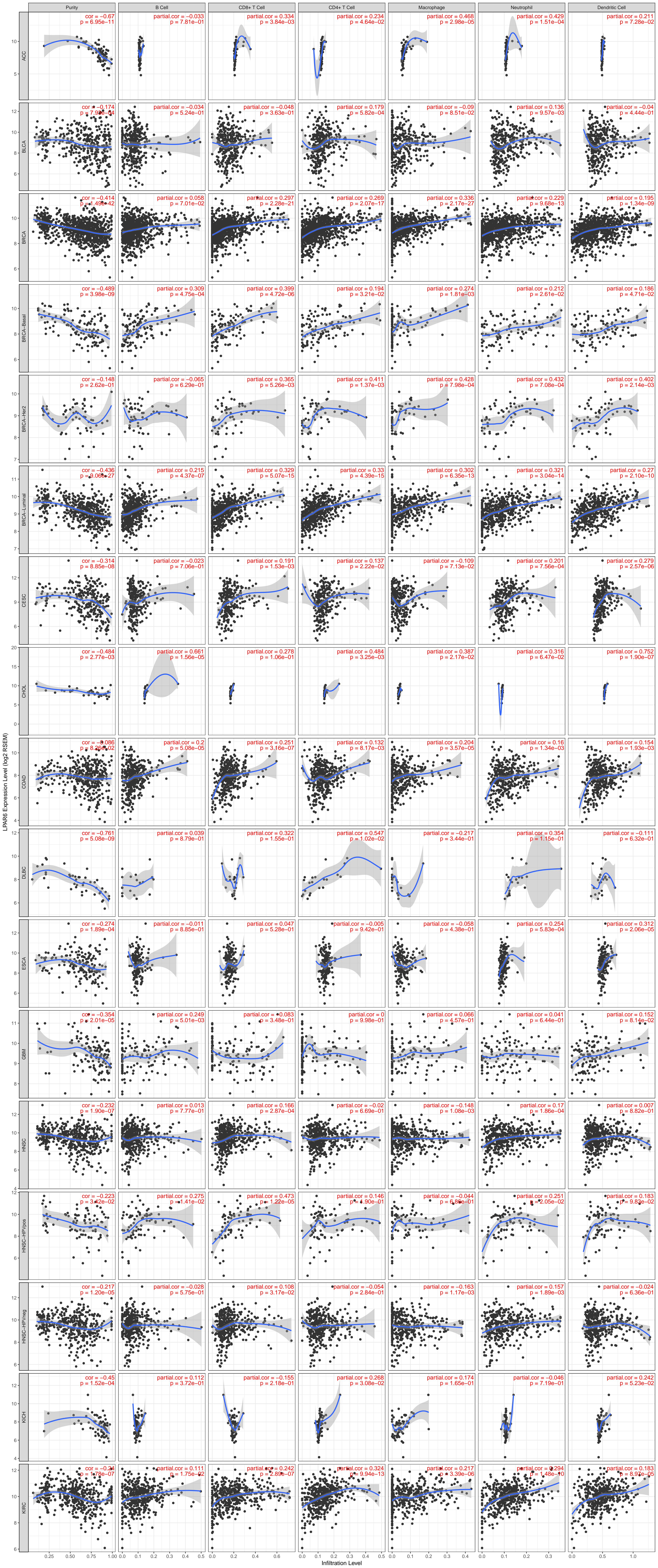

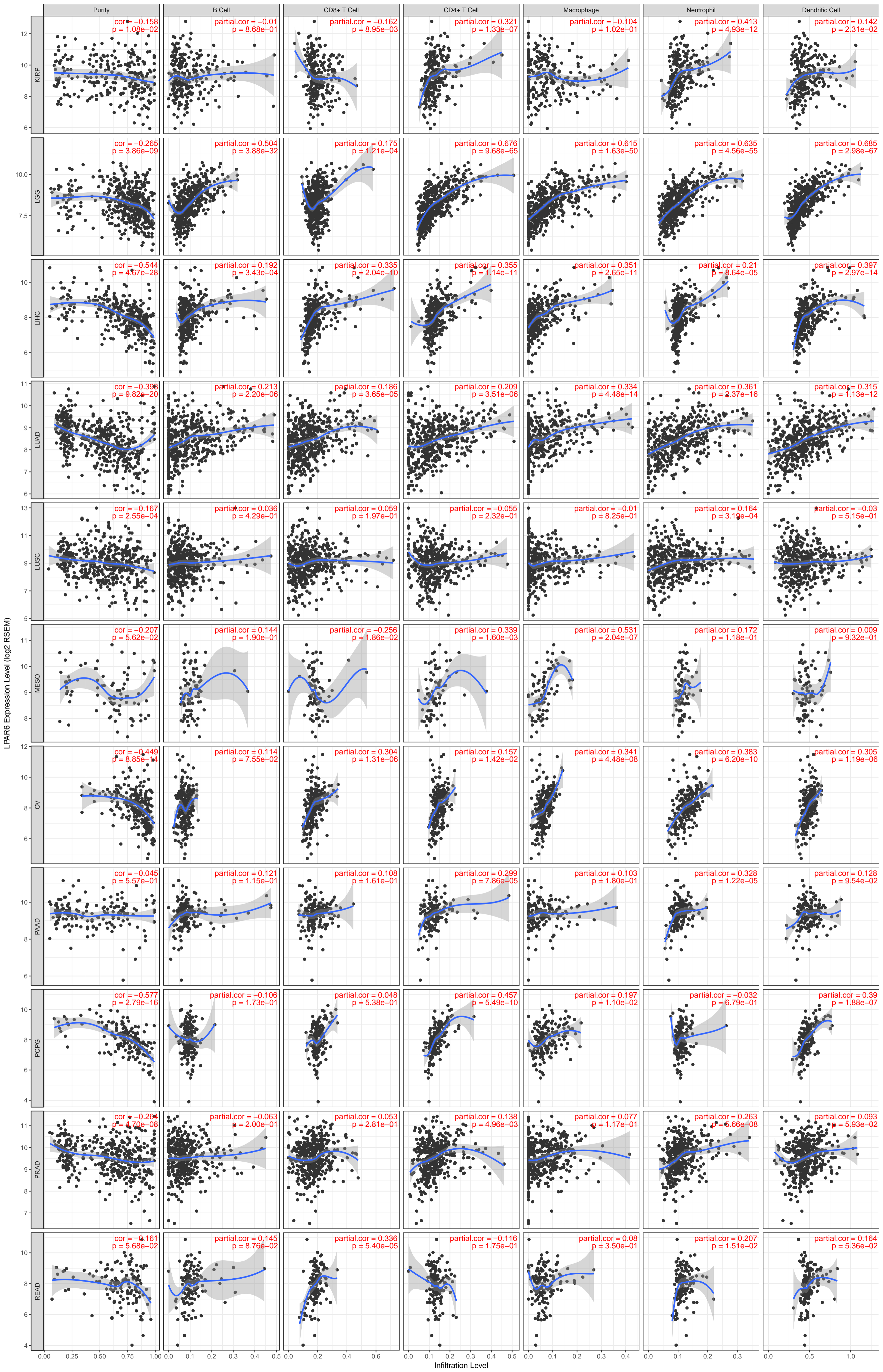

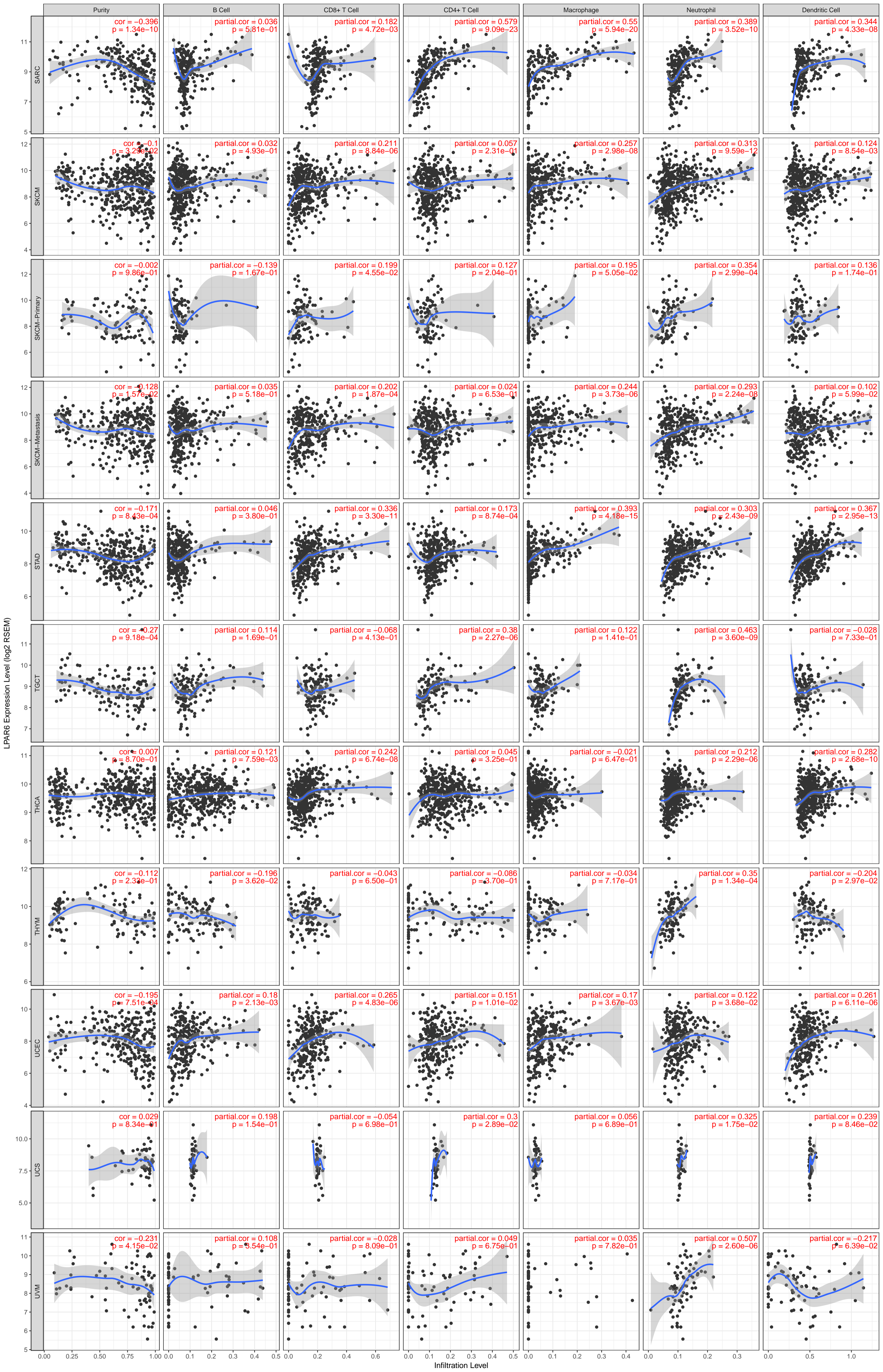

Supplement: Supplementary file 1 [file ijerph-18-11038-s001.zip › ijerph-1339832-supplementary/SI-Fig S3_R2.pdf]
